# Beyond the veil of duality—topographic reorganization model of meditation

**DOI:** 10.1093/nc/niac013

**Published:** 2022-09-27

**Authors:** Austin Clinton Cooper, Bianca Ventura, Georg Northoff

**Affiliations:** Integrated Program of Neuroscience, Room 302, Irving Ludmer Building, 1033 Pine Avenue W., McGill University, Montreal, QC H3A 1A1, Canada; Mind, Brain Imaging and Neuroethics Research Unit, Institute of Mental Health Research, University of Ottawa, 1145 Carling Avenue, Ottawa, ON K1Z 7K4, Canada; Mind, Brain Imaging and Neuroethics Research Unit, Institute of Mental Health Research, University of Ottawa, 1145 Carling Avenue, Ottawa, ON K1Z 7K4, Canada; Mental Health Center, Zhejiang University School of Medicine, 866 Yuhangtang Road, Hangzhou 310058, China

**Keywords:** meditation, self, duality, nonduality, nonduality, DMN, CEN, alignment, neurophenomenological

## Abstract

Meditation can exert a profound impact on our mental life, with proficient practitioners often reporting an experience free of boundaries between a separate self and the environment, suggesting an explicit experience of “nondual awareness.” What are the neural correlates of such experiences and how do they relate to the idea of nondual awareness itself? In order to unravel the effects that meditation has on the brain’s spatial topography, we review functional magnetic resonance imaging brain findings from studies specific to an array of meditation types and meditator experience levels. We also review findings from studies that directly probe the interaction between meditation and the experience of the self. The main results are (i) decreased posterior default mode network (DMN) activity, (ii) increased central executive network (CEN) activity, (iii) decreased connectivity within posterior DMN as well as between posterior and anterior DMN, (iv) increased connectivity within the anterior DMN and CEN, and (v) significantly impacted connectivity between the DMN and CEN (likely a nonlinear phenomenon). Together, these suggest a profound organizational shift of the brain’s spatial topography in advanced meditators—we therefore propose a topographic reorganization model of meditation (TRoM). One core component of the TRoM is that the topographic reorganization of DMN and CEN is related to a decrease in the mental-self-processing along with a synchronization with the more nondual layers of self-processing, notably interoceptive and exteroceptive-self-processing. This reorganization of the functionality of both brain and self-processing can result in the explicit experience of nondual awareness. In conclusion, this review provides insight into the profound neural effects of advanced meditation and proposes a result-driven unifying model (TRoM) aimed at identifying the inextricably tied objective (neural) and subjective (experiential) effects of meditation.

## Introduction

### Nondual awareness and the self

What is the experience of one’s seemingly real boundaries of a separate self dissolving into a state of unity with the world, that is, a lack of separation between self and other? One term that aids to describe this phenomenon is “nondual awareness” (Josipovic 2014, 2016, 2019, 2021; Berman and Stevens 2015). A description of nondual awareness has been proposed as “a background awareness that precedes conceptualization and intention and that can contextualize various perceptual, affective, or cognitive contents without fragmenting the field of experience into habitual dualities” (Josipovic 2013). Developing an understanding of the neuro-phenomenal mechanisms (Northoff and Zilio 2022) mediating the explicit experience of nondual awareness (Josipovic 2021) is the goal of this review.

Given that nondual awareness entails a radical change in the experience of the self with respect to others and the environment, we postulate that the self is not homogenous, rather it includes various layers, and that nondual awareness is signified by a radical shift in the relationship among them, both neuronally and experientially. To address this, we consider Qin *et al.*’s (2020) neural model, which subdivides self-processing into three intimately connected and spatially nested levels with different topographic extensions in the brain: interoceptive-processing, exteroceptive processing, and mental-self-processing.

**Figure 1.**
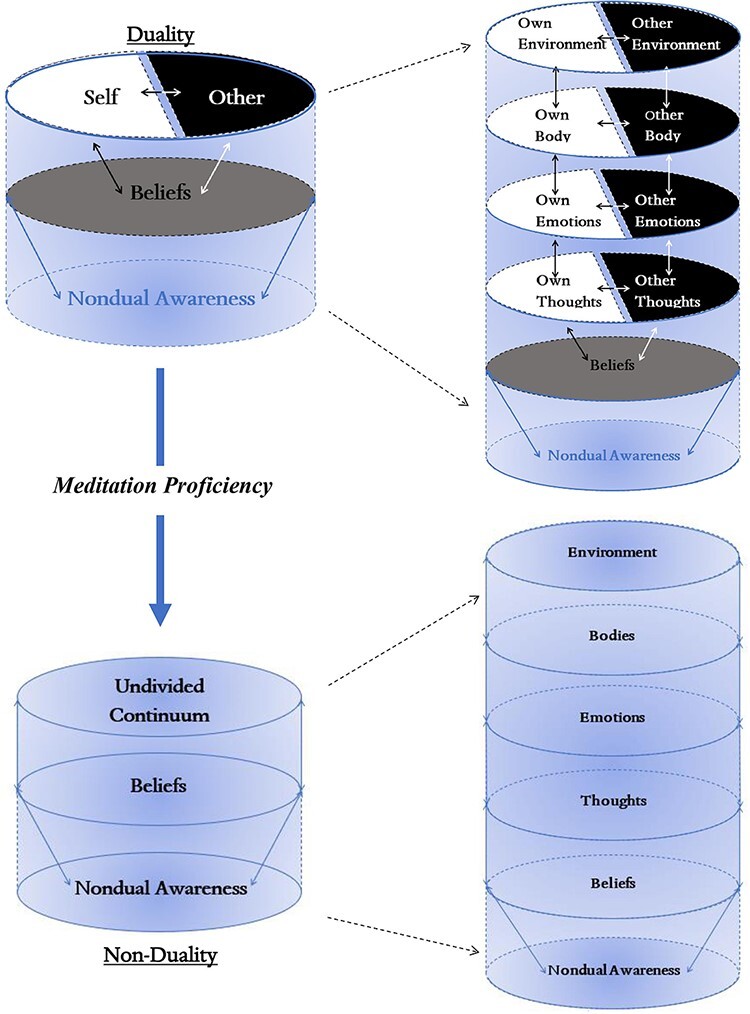
A visual depiction of nondual awareness’s relationship with meditation proficiency. Nondual awareness is necessary for all concepts of “self” and “other” to form, although these conceptualizations that are deeply linked with beliefs (see Box 3: relationship of TRoM to predictive coding, active inference, and psychedelics) are a hindrance to the explicit experience of nondual awareness itself. The text “Nondual Awareness” transition from light to dark to represent the shift from implicit to explicit experience. Though nondual awareness is always present, as represented by the light background/space that is preliminary to all levels and distinction that are fueled by beliefs

**Box 1.** TRoM’s relationship with the temporo-spatial theory of consciousness (TTC)

The TTC conceives consciousness as a basic temporo-spatial phenomenon: temporal dynamic and spatial topography are supposed to be shared by both neural and mental/phenomenal levels—the nondual topography on the brain’s neural level surfaces then as nondual experience on the phenomenal or experiential level. The TRoM thus lends further support to the TTC from the viewpoint of an altered state of consciousness, namely proficient meditation. Inextricably tied to the scale-free and nested spatiotemporal structure of the brain is the manner in which the brain can align its spatiotemporal activity to the environment (Northoff and Zilio 2022), something which we posit is fundamental to the explicit experience of nondual awareness. In order to align with the environment, there must both be a sufficient spatiotemporal repertoire (i.e. a sufficient range of functioning intrinsic neural timescales), and these timescales must be functionally aligned (something that is likely impacted during mind-wandering and definitely impacted in states of sedation and anesthesia). By promoting subjects to become increasingly aware of thoughts, emotions, bodily sensations, and environmental activity in a passive manner (depending on the specific meditation type) without yet explicit differentiation between inside/self and outside/other, meditation techniques support the alignment between brain, body, and environment—this allows for the direct experience or perception that all content arises within the shared space of nondual awareness. **Nestedness** Through increased meditation proficiency, we experience ourself no longer as dual to nonself but as more nested and integrated and thus connected with body and environment, e.g., temporo-spatial nestedness (see temporo-spatial alignment and nestedness as two key mechanisms in the TTC) (Northoff and Huang 2017, Northoff and Zilio 2022). **Alignment** This alignment (transition from implicit to explicit) of the scale-free spatiotemporally nested layers of self (nondual awareness) is posited as the link between the self and nondual awareness. We must be clear that the self in this case is not merely restricted to cognitive phenomenon and is more accurately viewed as the connection between brain, body, and environment. An effective analogy to nondual awareness proposed by Josipovic (2021) is that of a mirror: which is necessary for all content to be seen, though it is never affected by the contents themselves.

**Box 2.** Relationship of TRoM to other neuro-phenomenological models of meditation

**Fronto-parietal central precuneus network** (Josipovic 2021): The fronto-parietal precuneus network model is based on findings that suggest that the central precuneus is capable of functionally connecting with both intrinsically oriented (DMN) and extrinsically oriented (CEN) networks and thus “corresponds to a major function of nondual awareness by increasing the integration of intrinsic self-related and extrinsic environment-related aspects of experience” (Josipovic 2021, see also Josipovic *et al*. 2012; Josipovic 2014). This hypothesis is well in accordance with the TRoM as stated here. Both emphasize the reorganization of the relationship between the CEN and the DMN. The TRoM extends beyond the fronto-parietal hypothesis by emphasizing the longitudinal transformation between the DMN and CEN and how this relates to meditation proficiency and the layers of self-processing (mental, exteroceptive, and interoceptive). That renders possible to directly link topographic reorganization on the neural level of the brain with the topographic reorganization of the three layers of self, that is, the shift from mental dominant self-processing to an intero-exteroceptive dominant self-processing. The TRoM therefore allows to specify and extend the mechanisms mediating the shift from dual to nondual awareness in our consciousness, not only with measures of connectivity, but more dynamic measures of nestedness and alignment (see Northoff and Zilio 2022). **Adaptive coding net/adaptive workspace** (Raffone and Srinivasan 2009): The adaptive coding net model postulates that the awareness that meditation (primarily OMM forms) gives access to can be represented by an adaptive coding net (neurons especially located in the anterior PFC), which simultaneously links to multiple brain maps in parallel. This likely yields a large repertoire of potential network patterns available at any time. They further suggest that advanced meditators can elicit increasingly complex firing patterns, thus allowing the brain to more accurately reflect the changing patterns of body and environment. The TRoM is well in accordance with this hypothesis. The relative increase in the intero-exteroceptive self and its underlying neural correlates makes possible an enhanced capacity to stochastically match with the intero-exteroceptive input patterns from body and environment—the coding of these inputs may thus be more adaptive and less disturbed by the top-down predictions of the mental self. Future studies are warranted to add the temporal and thus dynamic dimension like the intrinsic neural timescales (Wolff *et al*. 2022, Golesorkhi *et al*. 2021a,b): we assume a broader dynamic range (Irrmischer *et al*. 2018) to characterize meditation, which may increase the capacity for more adaptive (en)coding of bodily and environmental inputs. Moreover, topographic reorganization goes along with more spatially extended global synchrony as, for instance, the negative relationship of DMN and CEN is replaced by more positive synchrony. Whether such increased synchrony extends to the whole brain thus amounting to a true global synchrony remains to be investigated in the future though. The data discussed here do indeed suggest less difference and more synchrony between unimodal and transmodal regions’ functional connectivity—given that functional connectivity is phase- and thus synchrony-based (Huang *et al*. 2015), this would be well in line with the assumption of global synchrony.
**Minimal phenomenal experience** (Metzinger 2020): The model of minimal phenomenal experience (MPE) as posited by Metzinger (2020) states that MPE is an inner representation of tonic alertness, which can co-exist with many other forms of conscious contents. It is further said it is a nonegoic form of self-modeling, which is mostly unnoticed since it functions as the transparent model of an abstract space in which other contents unfold over time. Predictive Bayesian modeling, within the brain, of the functional property of tonic alertness is seen as a primary dimension of MPE.
The TRoM agrees and disagrees with model of MPE. It agrees that there is less egoic self-modeling as this corresponds to the decrease in the mental self. At the same time, nonegoic self-modeling increases as manifested in the relative increase of the intero-exteroceptive self-processing. The TRoM thus specifies and further supports the shift from egoic to nonegoic self-modeling as well as the tacit assumption that meditation still involves a self or self-modeling albeit a different sense of self than the one most commonly associated with normal waking state in which nondual awareness is implicit. The TRoM disagrees with a more tacit assumption of the MPE, namely its implicit presupposition that experience is limited and constrained to the head and, at best, the body. This is clearly different in TRoM. Rather than being neuro-cognitive (or neuro-phenomenal), the TRoM is an explicit neuro-ecological model of meditation: it shifts the explicit focus of our experience toward our alignment to the bodily and especially environmental context. Such temporo-spatial alignment is a key component of the TRoM, which distinguishes it from the MPE and, as we would see, also from all other meditation approaches as well as from most consciousness theories (except the TTC; Box 1). Only by taking into view this neuro-ecological component, the nondual nature of experience beyond the self-environment dichotomy can be understood. Nondual awareness shows us in a paradigmatic way that, more generally, consciousness is strongly neuro-ecologically based albeit it commonly remains more tacit or implicit in our experience.

**Box 3.** Relationship of TRoM to predictive coding, active inference, and psychedelics

How does the TRoM relate to predictive coding? Laukkonen and Slagter (2021) state that during regular thinking (i.e., mind-wandering) one continuously yields top-down predictions (Pezzulo *et al*. 2021). By increasing the depth of meditative states, the frequency of predictions decreases such that one experiences the incoming inputs and events without prior appraisal. This leads to a recognition of nondual awareness, in which the predictive hierarchy is flattened and “the awareness inherent in experience becomes the foreground” (Laukkonen and Slagter 2021). In Laukkonen and Slagter’s model, they propose that FAM reduces mental or cognitive self-processing and brings the meditator into a more direct and embodied mode of being. Then, going further into the present-moment experience, OMM brings the meditator into a state of nonjudgmental open and bare experience, in a “sensing without appraisal” state (Laukkonen and Slagter 2021). They do not mention NDA meditation itself, which is free of intentional effort, directly aimed at identifying a reflexive awareness, and can be seen as primarily context-oriented, with nondual awareness as the primary context of experience as opposed to attending to the specifics of experience (Josipovic 2012). This discovery of the reflexive and choiceless awareness would be the form of awareness that is freest from predictive modeling since it is most simply the discovery of the space in which the predictions occur, like how a mirror cannot predict what it will reflect, neither can nondual awareness. Empirically, meditation has repeatedly shown evidence of decreased predictive coding. Evidence of the described gradual inhibition of Bayesian predictive modeling has been found in some studies. Valentine and Sweet (1999) found that OMM meditators were less biased by past temporal regularities than FAM meditators. These results may suggest that the OMM meditators develop less strong temporal expectations that biased subsequent perception, potentially because open awareness is further along the progression of awareness. More evidence of the inhibition of predictive modeling that occurs through meditation can be found in Hanley and Garland (2019), in which the authors show that mindfulness training can reduce Pavlovian conditioning. This is very well compatible with our observation of the decrease in the mental self and its underlying regions, DMN. The mental self may no longer yield as strong top-down predictions for especially the lower layer of self, the intero-exteroceptive self. The latter is thus less shaped by the mental self and its duality of self-nonself. Instead, the intero-exterocetpive self may more strongly shaped by bottom-up processing from the relationship of self and environment rather than by top—this allows to focus more on present-moment experience and “sensing without appraisal.” Additionally, the more spontaneous engagement with the world in meditation is also understandable through the perspective of criticality. Systems at a high level of criticality are at a transition point between different states and show scale-free similarity, long-range correlations and critical slowing (Chialvo 2014; Carhart-Harris and Friston 2019). Since meditation experience has been shown to significantly increase the long-range temporal correlations, a measure of criticality (Irrmischer *et al*. 2018), it is quite probable that meditation, just like psychedelics, reduces the impact of previously formed generative models, allowing for a more spontaneous and free-flowing interaction with the world. The increase in long-range correlations means that more distant regions can be linked to each other and thus synchronize in a positive way to a higher degree—this is not only in tune with the reported findings but also with the reduced degree of the brain’s dual topographic organization including its replacement by nondual topography in proficient meditation. Finally, psychedelics have shown similar neural effects as meditation experience: decreased DMN activity (Muthukumaraswamy *et al*. 2013; Tagliazucchi *et al*. 2014; Carhart-Harris *et al*. 2016), decreased anterior-posterior DMN connectivity (Carhart-Harris *et al*. 2012a), increased connectivity between the DMN and task-positive networks (Carhart-Harris *et al*. 2012b), and increased global neural- connectivity (Carhart-Harris *et al*. 2016; Tagliazucchi *et al*. 2016). Together with analogous experience of nonduality (Carhart-Harris and Friston 2019), these data provide further evidence of profound topographic reorganization in meditation leading to the experience of nondual awareness (Northoff *et al*. 2020b). Furthermore, a model for a phenomenon often experienced during psychedelic experiences, which is related to the reduction of mental-self-processing, referred to as ego-loss, has been termed: relaxed beliefs under psychedelics (REBUS) (Carhart-Harris and Friston 2019). In this state, the brain is said to relax the temporally bound top-down processing in exchange for an increased sensitivity to bottom-up processing, hence allowing for a new engagement with the world (Carhart-Harris and Friston 2019; Deane 2020). This again is well compatible with the TRoM. The decrease in DMN–CEN dichotomy and the decrease in mental self (and its underlying regions) strongly suggest decreased top-down predictions and, as a consequence, more relaxed beliefs. Hence, the TRoM and especially the topographic shift from mental to intero-exteroceptive self are well compatible with the REBUS model in psychedelics. Future studies are warranted, though, to better flesh out similarities and differences of meditation and psychedelics.

### What is meditation and how does it relate to nondual awareness?

The term meditation has been referred to as inadequate, considering the extremely wide range of practices that it refers to (Lutz *et al.* 2007). Nevertheless, there is a common purpose between all practices: to decrease psychological suffering through regulatory (e.g. attentional, emotional, proprioceptive, and interoceptive) processes. Indeed, according to Buddhist traditions, there are a set of correctable defects that are the root cause of human suffering (Gethin 1998; Lutz *et al.* 2007). Generally speaking, a meditation practice can begin with an emphasis on focusing the mind on an object (such as the breath, a mantra, or an image), followed by an emphasis on the awareness of subjectivity itself, which then at the highest level of practice can progress to a disidentification from any subject or object and thus a realization of the fundamentally changeless and nondual (reflexive) awareness in which all changing contents of experience take place (Lutz *et al.* 2007).

The array of different meditation types has already been characterized by unique neural correlates, both structurally and functionally (Fox *et al.* 2016; Laukkonen and Slagter 2021). Despite the neural and psychological differences among the different meditation techniques, there are commonalities between meditation types that are related to the reduction of suffering: (i) monitoring of attention and some aspect of the present moment (Lutz *et al.* 2008; Lippelt *et al.* 2014; Delorme and Brandmeyer 2019), (ii) associated with trait increases in mindfulness (Kiken *et al.* 2015; Lemay *et al.* 2019; Tang and Tang 2020; Sousa *et al.* 2021) (i.e. awareness of both mental and environmental content), and (iii) commonly involve decreases in perceived stress (Winzelberg and Luskin 1999; Mohan *et al.* 2011; Chambers *et al.* 2016; Lemay *et al.* 2019). Or put most succinctly, “all meditations in one way or the other try to get you to live in the present” (Osho 2012).

Various brain imaging studies of meditation report changes in attention, memory, and emotional-related regions and networks of the brain (Brefczynski-Lewis *et al.* 2007; Brewer *et al.* 2011; Farb *et al.* 2012; Hasenkamp *et al.* 2012; Fingelkurts *et al.* 2015; Tang *et al.*  2015a; Fox *et al.* 2016; Travis and Parim 2017), but how this relates to the explicit experience of nondual awareness is a question left unanswered? The capacity to explicitly reach this core state of unity along with other preliminary meditative states is likely a matter of meditation proficiency, which has been correlated with the amount of meditative experience that one has cumulated (van den Hurk *et al.* 2010; Slagter *et al.* 2011; Lumma *et al.* 2015; Tanaka *et al.* 2015), rather than being strictly tied to the type of meditation that is practiced.

We must be clear that the unique functional and structural effects of distinct meditation types are evident (Fox *et al.* 2014, 2016; Valk *et al*. 2017), and we are thus operating under the assumption that all meditation types can lead to or at least contribute in the direction toward an experience of nondual awareness. With repeated evidence of highly proficient long-term meditators from different meditation backgrounds showing the capacity to transcend the self/other duality (Josipovic *et al.* 2012; Dor-Ziderman *et al.* 2013; Josipovic 2014, 2019; Ataria *et al.* 2015; Berman and Stevens 2015; Shiah 2016), we see this as a fair assumption. So, meditation’s orientation toward a present-moment awareness seems to diminish the magnitude of self-referential thinking, hence decreasing the perceived self-other duality (Farb *et al.* 2007; Lutz *et al.* 2015; Hasenkamp 2018; Millière *et al.* 2018; Bauer *et al.* 2019).

### Topographical reorganization model of meditation

Given the various spatial-topographic and temporal dynamic changes in the brain’s neural activity associated with meditation, we suppose that they translate into more or less corresponding spatiotemporal changes in our experience on the mental level. In other terms, the brain’s topographic reorganization between higher-order self-related regions [default mode network (DMN)] and lower-order nonself-related regions [central executive network (CEN)] translates into a more or less corresponding nondual reorganization of our experience—nonduality is shared by neural and mental levels as their “common currency” (Northoff and Lamme 2020a; Northoff *et al.* 2020b). We therefore postulate what we describe as “topographic reorganization model of meditation” (TRoM). The TRoM has commonalities to some of the most prominent neuro-phenomenological models of meditation, though it is unique in its own right (see Box 2 for more).

Our main aim is therefore to review recent studies probing the effect of meditation on the DMN and CEN as well as their relationship with each other. Given that the self and other/nonself are not experienced dichotomously within nondual awareness, we hypothesize that DMN activity is reduced and that the DMN–CEN relationship shifts from a negative to a positive connectivity (already proposed by Josipovic *et al.* 2012; Josipovic 2014), most likely during meditation itself but quite possibly sustained during the resting state (RS) of highly proficient meditators (see a single case report by Ataria *et al.* 2015). For that purpose, we review two main lines of imaging [functional magnetic resonance imaging (fMRI)] studies: those specific to the neural effects of meditation and those probing the interaction of meditation and self.

## Methods

### Search terms

The review focused on meditation-related changes in activation and connectivity from fMRI studies, including the self × meditation interaction. See Fig. 3 for a visual depiction of the search terms used in this review to gather the studies that were implemented.

### Meditation type categorization

It shall be mentioned that there is a large variety of different meditation practices that have been probed in fMRI research. In a recent meta-analysis, Fox *et al.* (2016) narrowed meditation into four general categories: focused attention meditation (FAM), mantra recitation meditation (MRM), open monitoring meditation (OMM), and loving-kindness and compassion meditation (LKM). In addition, Josipovic *et al.* (2012) illustrated that a unique form of meditation that has a very precise orientation toward experiencing nondual awareness (nondual awareness meditation) shows a distinct neural signature when compared to FAM and OMM, that is why it has been added here as a distinct form of meditation.

**Figure 2.**
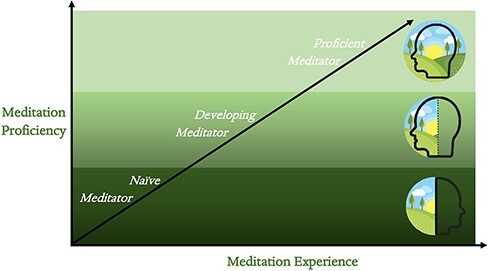
Practice of any form of meditation practice is seen as contributing toward the development of the capacity to explicitly experience nondual awareness. Meditation proficiency and experience are understood as correlated, although, this relationship is definitely not as straightforward as displayed since different meditation practitioners with the same amount of experience do not necessarily have the same subjective states during rest and meditation

The reason for which this review is very inclusive toward specific meditation practices is based on the viewpoint that the nondual state of awareness is obtainable through all forms of practice (Reddy and Roy 2018, 2019) and that it is consistent across all different meditation types as pointed out especially by Rodriguez-Larios *et al.* (2020) (see also Berman and Stevens 2015)(Fig. 2). This will serve to create a general framework around meditation and the self; yet, it is understood that this is a simplification that doesn’t fully take all individual meditation types specific to “neural fingerprints” into account. Further investigation into this phenomenon will be necessary to develop a more pedantic framework and see how each type of meditation is directly associated with progression toward the recognition of nondual awareness.

**Figure 3.**
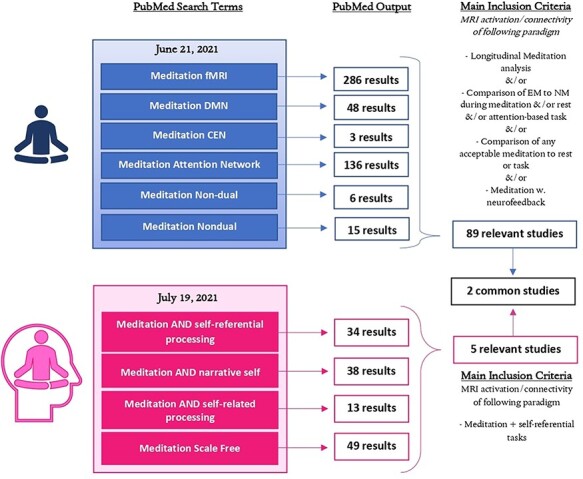
Flow chart of screening process for reviewed studies. Upper blue half represents the screening that was specific to meditation only, and the bottom pink half represents the screening that was specific to the interaction between meditation and the self

## Requirements for inclusion

### Meditation section

#### Inclusion criteria

Our main focus was on the spatial features (mainly fMRI—including activation and connectivity) of meditation. Any study that explicitly studied activation or connectivity measures from fMRI in a relevant comparison or correlational manner was included. Studies that reported on changes in these features either between varying levels of meditation experience (whether it be between different groups or between one group both before and after meditation training), between meditation and RS or a task, or a correlational analysis between meditation type or meditation experience and spatial features of interest were included. Group comparisons of meditation experience (both within- and between-group) were also included for measurements obtained during both RS and meditation.

**Table 1.** Studies that repeatably find decreased DMN activation because of meditation

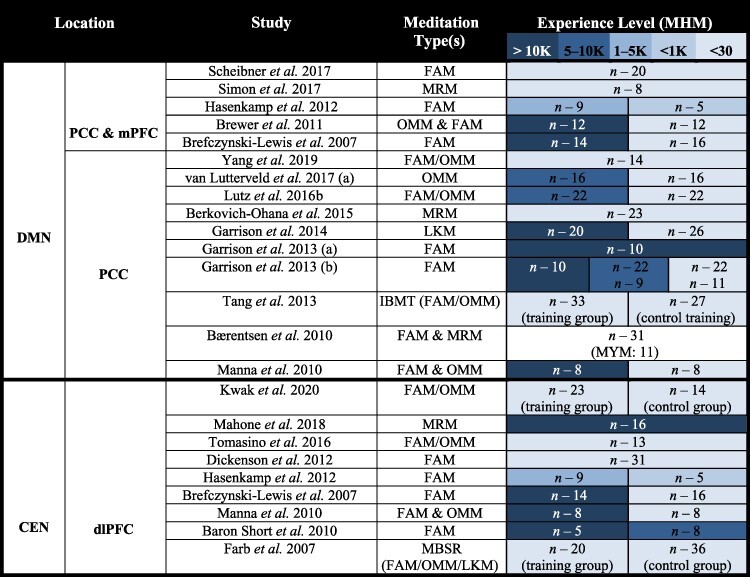

Supporting Information: Text within sections allocated to MHM is to specify either the differentiating factor between groups of similar experience level (e.g. meditation training vs relaxation training) or the level of meditation when mean hours of experience were not provided within the published paper.

Abbreviations: dorsolateral prefrontal cortex (dlPFC); integrative body-mind training (IBMT); mean hours of meditation (MHM); mean years of meditation (MYM); medial prefrontal cortex (mPFC); mindfulness-based stress reduction (MBSR).

### Borderline inclusion

- Studies that analyzed subjects with some form of mild-to-moderate psychological distress (e.g. social anxiety disorder, childhood neglect, and posttraumatic stress disorder) were included in this review, although they were not the primary focus of our attention.
- Studies that incorporated some form of neurofeedback during meditation were also included because of their direct capacity to correlate precise regional locations of neural activity with phenomenological experience.
- Studies that focused on meditation and its effect on task-based neural activity were only included if the task was attentionally based, as that can provide insight into how meditation experience leads to increased capacity to modulate where attention is placed.

#### Exclusion criteria

We did not include strictly morphological studies or studies in which their analysis focused on seed-based connectivity with regions not included in our primary focus of the DMN and CEN, event-related potentials, pain perception/modulation, emotional reaction to stimuli, emotional regulation, hypnosis, binaural beats, drug-induced activity, praying, and/or religious chanting (chanting in general was excluded).

Aside from these forms of analysis, the linear activation and connectivity analysis from electroencephalogram (EEG)/Magnetoencephalography (MEG) have been excluded from the main analysis of this review due to their lack of standardization affiliated with the signal processing techniques, which is likely tied to the results of extremely heterogenous nature (Deolindo *et al.* 2020).

The details of the different studies along with all of their relevant findings can be found in the Supplementary Material. Here, in the main text, we summarize the main and most consistent findings in order to streamline the significant complexity and variety of results and approaches in imaging studies on meditation. We did, although, integrate the meditation type and meditation experience levels of the study subject groups to decipher if these metrics are primary components, which determine or impact the results obtained.

### Meditation and self-section

In this section, we focused on studies that included in their experimental paradigm self-processing tasks. We did that in order to see how the different layers of self-processing change in proficient vs naïve meditators.

#### Inclusion criteria

We looked at studies about self and meditation, which included self-processing tasks in their experimental paradigm, to infer how self-processing changes by gaining experience in meditation.

Also in this section, we focused on the spatial domain (MRI and fMRI—including activation and connectivity). A study that analyzed subjects with a social anxiety disorder was included in this section of the review.

#### Exclusion criteria

We did not include studies that did not have any kind of self-referential processing task in their experimental paradigm and that focused on self-processing only in the discussion, as an interpretation. We did not include studies that focused on dynamic features (both fMRI and EEG/MEG). Like in the meditation section, here we excluded strictly morphological studies and event-related potentials.

## Results

### Network activity and connectivity

Regions from within the DMN and CEN [and also the salience network (SN)] proved to show the most promising and relatively replicable results throughout the range of reported findings; hence, their results are selected and cumulated for analysis. To disentangle the various measures used in different studies, we distinguish three main measures: network activation, network intra-connectivity, and network interconnectivity. These parameters are valuable when comparing different meditation techniques to each other as well as to the RS, along with comparing different subject groups with markedly different experience levels. Here, the primary regions that are focused are the posterior cingulate cortex (PCC), precuneus (PCu), and medial prefrontal cortex (mPFC) for the DMN; dorsal lateral prefrontal cortex (dlPFC) for the CEN; and the anterior cingulate cortex (ACC) and insula for the SN.

### Core network activity findings

#### Decreased posterior DMN activation

**Figure 4.**
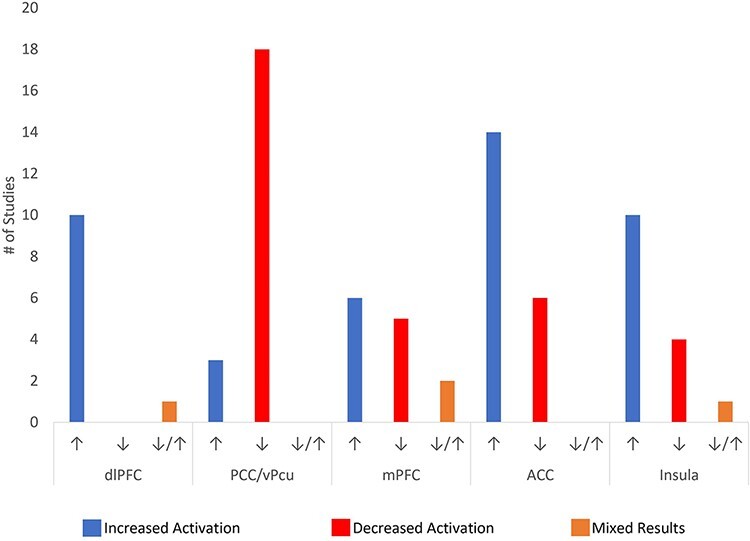
Tally of activations/deactivations of each primary ROI from reviewed articles

The DMN, and mainly the posterior DMN, consistently deactivates during a range of meditation practices (Table 1 and Fig. 4). Several forms of meditation have directly exhibited a greater DMN deactivation when compared to self-related focus (Farb *et al.* 2012), self-related tasks (Garrison *et al.* 2015) as well as other active tasks including a visual task (Ives-Deliperi *et al.* 2011) and finger tapping (Simon *et al.* 2017). This reduced DMN activity when comparing meditation to an active task was only seen for experienced meditators but not in naïve controls (Garrison *et al.* 2015). This signifies the progressive decline of DMN activity during meditation as being associated with meditation experience. Likewise, meditators show a significantly larger decrease of DMN activity than controls, when comparing meditation to the RS (Brewer *et al.* 2011; Garrison *et al.* 2015). As evidenced by, deactivation of the PCC was the most replicated finding when it comes to meditation, this shows the robustness of this finding.

See the Supplementary Material for all DMN activity-related findings.

#### Increased CEN activation

The CEN (primarily the dlPFC) often shows an increase in activation during meditation, with more advanced meditators showing the capacity to keep it more consistently activated (Short *et al*. 2010). This increase in activation is especially occurrent during focused awareness meditation, yet it is also quite evident in other more open forms of meditation (see Table 1). Yet the trend associated with CEN activation may be nonlinear with respect to meditation proficiency. Significantly, Brefczynski-Lewis *et al.* (2007) found that the group of meditators with the middle most level of meditation experience elicited the greater dlPFC activity during meditation compared to both naïve meditators as well as the most experienced group of meditators. This is significant considering that the most experienced group of meditators had an average of 44 000 h of experience, while the intermediate meditation group had approximately 19 000 h of experience. Most other studies do not include participants with experience levels greater than 19 000 h (likely because it is a rarity to find subjects with this magnitude of experience), meaning that this study was able to effectively analyze a level of meditation experience commonly overlooked.

This phenomenon alludes to an inverse U-shape trend of CEN activation associated with increasing meditation proficiency, which is in line with previous commentaries on how meditation follows a trajectory from being effortful to effortless (Tang *et al.* 2015b—see the Discussion for more on this point). That is also in line with the proposition of an evolved default state where open forms of meditation are neuronally very similar to the RS (Manna *et al.* 2010) and where large-scale networks are increasingly positively connected (Brewer *et al.* 2011) (see below for network connectivity). It should be noted that this is not yet conclusive evidence, and more research is required to assess if this nonlinear neural phenomenon is robust and how it is related to the subjective transformation of increased meditation proficiency.

See the Supplementary Material for all CEN activity-related findings.

#### Split Findings for mPFC Activation/deactivation

Meditation’s impact on mPFC activity provides the least consistent finding out of all Region of interests (ROIs) that were assessed in our review (Table 2). We attempted to find any trends regarding mPFC activity level, precise location within the mPFC, meditation type, and experience level, but to no avail. Interestingly, meditation-related increased mPFC activity was found when contrasting highly experienced meditators to naïve meditators, testing longitudinal effects of meditation training, and also when directly comparing neural activity during meditation to rest. Meditation-related decreases of mPFC activation, although, were not found when directly assessing the longitudinal impact of meditation, primarily in the RS. Whether this suggests increased meditation proficiency is not associated with reduced RS mPFC activity is highly uncertain and worthy of investigation. Additionally, deactivations of the mPFC during meditation seemed increasingly relevant to MRM (Engström and Söderfeldt 2010; Xu *et al.* 2014). The only other study that found an increased activation of the mPFC during meditation utilized a contrast between FAM and mental arithmetic (Hölzel *et al.* 2007); this is not an ideal contrast for our purposes since mental arithmetic in itself requires focused and deliberate attention, unlike the RS. Meanwhile, mPFC deactivations during meditation were found during FAM (Brewer *et al.* 2011; Scheibner *et al.* 2017) and LKM (Brewer *et al.* 2011) when compared to the RS (Brewer *et al.* 2011) and mind-wandering (Scheibner *et al.* 2017). The relationship of meditation-related mPFC activation specificity is still a question that requires further analysis.

**Table 2.** Studies that find altered activation in the anterior DMN (mPFC) as a result of meditation

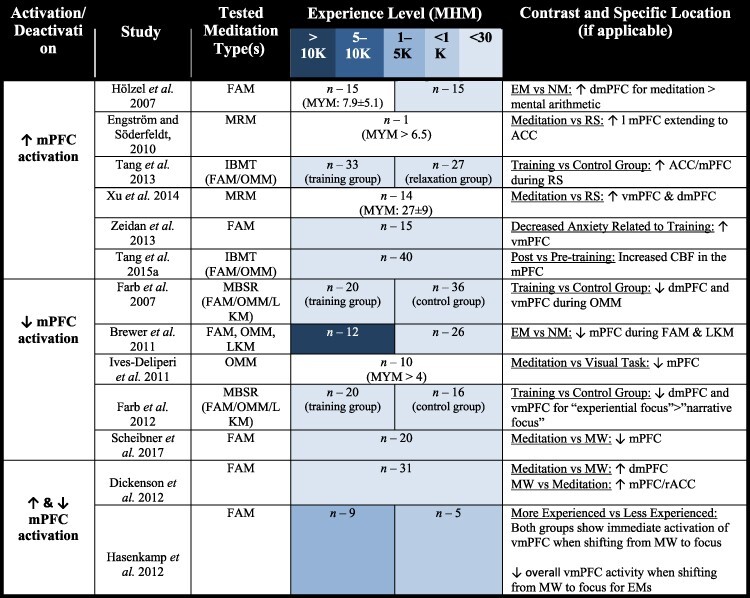

Supporting Information: Text within sections allocated to MHM is to specify either the differentiating factor between groups of similar experience level (e.g. meditation training vs relaxation training) or the level of meditation when mean hours of experience were not provided within the published paper.

Abbreviations: anterior cingulate cortex (ACC); dorsomedial prefrontal cortex (dmPFC); experienced meditators (EM); integrative body–mind training (IBMT); mindfulness-based stress reduction (MBSR); medial prefrontal cortex (mPFC); mean hours of meditation (MHM); mean years of meditation (MYM); mind-wandering (MW); naïve meditators (NM); nondual awareness meditation (NDA); ventromedial prefrontal cortex (vmPFC).

**Figure 5.**
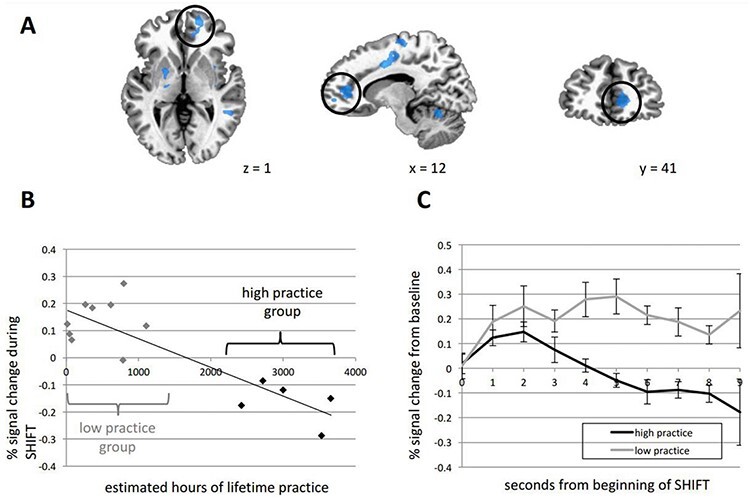
“Practice time effects. (A) Several clusters that were negatively correlated with practice time during the SHIFT phase. The ventromedial PFC cluster that was examined in B and C is circled. (B) Scatter plot of the relationship between practice time and fMRI signal in the ventromedial PFC cluster. Participants with high and low practice time are clearly segregated. (C) Time courses from the ventromedial PFC cluster were extracted, and Hemodynamic response functions (HRFs) were calculated from the onset of the SHIFT phase for each subject. Percent signal change (from mind-wandering, mean ± s.e.m.) over time is plotted for high (n = 5) and low (n = 9) practice participants. The BOLD response is significantly reduced in high practice compared to low practice participants across the modeled time series. *Main effect of group over time by repeated-measures Analysis of variance (ANOVA), p = 0.010.” All sourced directly from Hasenkamp and Barsalou (2012) with slight modification to the caption (changed “MW” to “mind-wandering”). SHIFT represents a shift from mind-wandering to focused attention

Although the meditation-related mPFC activity changes are difficult to make sense of, there have been studies that found a nonlinear mPFC activity phenomenon associated with meditation. Specifically, Hasenkamp and Barsalou (2012) found that there was a nonlinear relationship between activity of the ventromedial prefrontal cortex (vmPFC) and the meditation experience level during a FAM paradigm (Fig. 5). The vmPFC was shown to activate when meditators transitioned from a state of mind-wandering back to the meditation object of focus (in this case the breath). Although, meditation practitioners with a mean of less than 1200 h of meditation showed prolonged vmPFC activation following this shift, whilst meditators with approximately double the meditation experience did not. In fact, the most experienced meditators had an overall negative vmPFC % signal change, where the magnitude of signal decrease was correlated with meditation experience.

Additionally, Dickenson *et al.* (2012) showed that mind-wandering was associated with increased activity in a cluster sharing the mPFC and rACC, when contrasted with neuronal activity during meditation. Although, meditation, when contrasted with mind-wandering, was associated with increased dmPFC activity, likely an illustration of the multifaceted nature of the mPFC region of the brain.

We further assessed the mPFC-specific findings to see if there is any pattern of coactivation/deactivation with other ROIs (Table 3). Specifically, we were interested to see how the significant change in meditation-related mPFC was associated with the PCC, the CEN, and the SN.

**Table 3.** Coactivation/deactivation findings associated with meditation related changes of mPFC activity

mPFC finding	Coactivation/deactivation ROI		Tested meditation type(s)	Study and findings
↑ mPFC activation	PCC/vPCu	↑ Activation	MRM	Xu *et al.* (2014): increased vmPFC, dmPFC, and PCC activity during nondirective MRM
		↓ Activation	IBMT (FAM/OMM)	Tang *et al.* (2013): following meditation training there was increased ACC/mPFC activity along with decreased PCC activity during RS
			FAM	Zeidan *et al.* (2013): increased vmPFC along with decreased PCC correlate with decreased anxiety as a result of meditation practice
	CEN	↑ Activation	IBMT (FAM/OMM)	Tang *et al.* (2013): following meditation training there was increased ACC/mPFC along with increased IFG/vlPFC activity during RS
			MRM	Xu *et al.* (2014): increased vmPFC, dmPFC, and IPL activity during nondirective MRM
	Insula	↑ Activation	FAM	Zeidan *et al.* (2013): increased vmPFC along with increased anterior insula correlate with decreased anxiety as a result of meditation practice
			IBMT (FAM/OMM)	Tang *et al.* (2015a): increased CBF in mPFC and vACC following FAM/OMM training
	ACC	↑ Activation	FAM	Hölzel *et al.* (2007): increased dmPFC and rACC activity when comparing Ems to NMs for a meditation vs mental arithmetic contrast
			MRM	Engström and Söderfeldt (2010): increased mPFC extending to ACC activity during MRM compared to RS
			FAM	Zeidan *et al.* (2013): increased vmPFC and ACC activity along with correlate with decreased anxiety as a result of meditation practice
			IBMT (FAM/OMM)	Tang et al. (2015a): increased CBF in mPFC and insula following FAM/OMM training
↓ mPFC activation	PCC	↓ Activation	FAM, OMM, and LKM	Brewer *et al.* (2011): decreased mPFC and PCC activity during FAM and LKM
			OMM	Ives-Deliperi *et al.* (2011): decreased mPFC and PCu activity for OMM vs a visual task
			FAM	Scheibner *et al.* (2017): decreased mPFC and PCC for FAM vs mind-wandering
	CEN	↑ Activation	MBSR (FAM/OMM/LKM)	Farb *et al.* (2007): decreased dmPFC and vmPFC along with increased dPFC and inferolateral PFC activity for EMs vs NMs during OMM
			MBSR (FAM/OMM/LKM)	Farb *et al.* (2012): decreased dmPFC and vmPFC along with increased lPFC activity for EMs vs NMs during “experiential focus” compared to “narrative focus”
	Insula	↑ Activation	MBSR (FAM/OMM/LKM)	Farb *et al.* (2007): decreased dmPFC and vmPFC along with increased insula activity for EMs vs NMs during OMM
			OMM	Ives-Deliperi *et al.* (2011): decreased mPFC and insula activity for OMM vs a visual task
			MBSR (FAM/OMM/LKM)	Farb *et al.* (2012): decreased dmPFC and vmPFC along with increased insula activity for EMs vs NMs during “experiential focus” compared to “narrative focus”
	ACC	↑ Activation	OMM	Ives-Deliperi *et al.* (2011): decreased mPFC and vACC activity for OMM vs a visual task
↑ & ↓ mPFC activation	PCC	↑ Activation	FAM	Dickenson *et al.* (2012): increased mPFC/rACC, dmPFC, PCC, and PCu activity during mind-wandering compared to FAM
			FAM	Hasenkamp *et al.* (2012): increased mPFC and PCC activity during mind-wandering
	Insula	↑ Activation	FAM	Dickenson *et al.* (2012): increased dmPFC and mid insula activity during FAM compared to mind-wandering
	ACC	↑ Activation	FAM	Dickenson *et al.* (2012): increased dmPFC and dACC activity during FAM compared to mind-wandering

Abbreviations: anterior cingulate cortex (ACC); dorsomedial prefrontal cortex (dmPFC); experienced meditators (EM); integrative body–mind training (IBMT); mantra recitation meditation (MRM); mean hours of meditation (MHM); medial prefrontal cortex (mPFC); mind-wandering (MW); mindfulness-based stress reduction (MBSR); naïve meditators (NM); ventromedial prefrontal cortex (vmPFC).

### Core network connectivity findings

#### Decreased posterior DMN intra-connectivity, increased anterior DMN intra-connectivity, and decreased posterior-anterior DMN intra-connectivity

A highly replicated result is that of a decoupled intra-network DMN connectivity (Table 4). In this vein, meditation has been shown to exhibit a decrease in operational synchrony within the posterior DMN (Fingelkurts *et al.* 2015) as well as a reduced nodal connectivity following meditation training (Cotier *et al*. 2017). Although meditation experience is associated with an increased anterior DMN connectivity (Jang *et al.* 2011; Fingelkurts *et al.* 2015; Panda *et al.* 2016; van Lutterveld *et al.* 2017a; Kwak *et al.* 2019). Garrison *et al.* (2014) indeed found that naïve meditator PCC was increasingly connected to other cortical midline structures of the DMN during LKM, whilst more experienced meditators displayed a PCC with functional connectivity patterns expanded beyond the DMN, especially to the inferior frontal gyrus. Also, since the anterior DMN activation during self-appraisal is significantly larger for experienced as opposed to nonexperienced meditators (Lutz *et al.* 2016b), this region is likely more affiliated with observing mental phenomenon (Fingelkurts *et al.* 2015).

**Table 4.** Dominantly repeated DMN & CEN intra-connectivity patterns associated with meditation

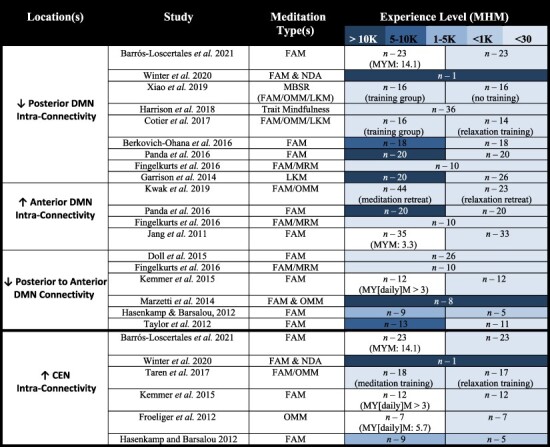

Supporting Information: Text within sections allocated to MHM is to specify either the differentiating factor between groups of similar experience level (e.g. meditation training vs relaxation training) or the level of meditation when mean hours of experience were not provided within the published paper.

Abbreviations: mindfulness-based stress reduction (MBSR); mean hours of meditation (MHM); mean years of meditation (MYM); nondual awareness meditation (NDA).

It should be stated that some outliers from these trends do exist in the literature. Avvenuti *et al.* (2020) found increased RS functional connectivity between the PCC, precuneus, and superior parietal lobule for a group that trained in transcendental meditation (a form of MRM) for 3 months. Additionally, Bauer *et al.* (2019) found that experienced meditators had increased functional connectivity between the PCC and the right angular gyrus (another region of the DMN). This being said, the dominant trends of the findings to date are represented in Table 4.

#### Increased CEN intra-connectivity

The CEN consistently shows to become more intra-connected, which when combined with the decreased interconnectivity of the DMN highlights a global topographic reorganization (see Table 4). This increased CEN intra-connectivity may be a result of the enhanced cognitive/top-down control (Froeliger *et al.* 2012; Taren *et al.* 2017; Winter *et al.* 2020; Barrós-Loscertales *et al.* 2021), which is granted through meditation experience and is likely tied to the meditators increased capacity to induce activity in the CEN in order to quiet the activity of the DMN (primarily the PCC).

#### Altered DMN to CEN interconnectivity

**Figure 6.**
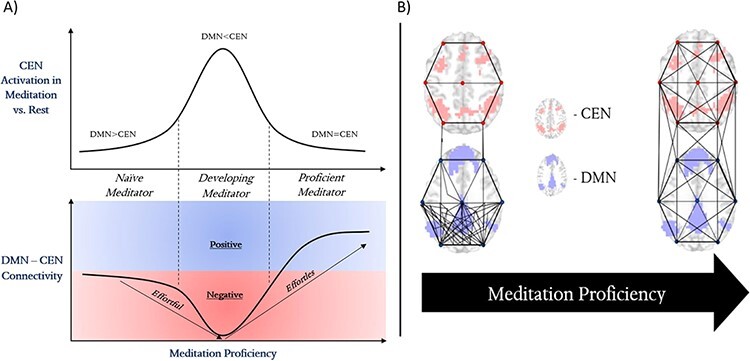
A visual depiction of the transition from effortful to effortless meditation. (A) The practicing meditator learns to maintain focused attention through CEN activation; although once a certain level of proficiency (a transformed default mode) is reached, there is no longer a need for extensive CEN activation, and there is an extended default mode in which DMN and CEN can be coactive. Coinciding with CEN activity is the connectivity between the DMN and CEN networks of the brain: effort is needed to suppress DMN hyperactivity and activate the CEN (negative connectivity/anticorrelation), which evolves to a coactivation (positive connectivity) of the two networks. This coactivation is affiliated with experience of explicit nondual awareness. (B) The transition from dominant DMN, to dominant CEN, and finally to co-active DMN and CEN is affiliated with global connectivity changes. Primarily, the posterior DMN’s intra-connectivity is decreased, both anterior DMN’s and CEN’s intra-connectivity is increased, and the interconnectivity between the DMN and CEN is increased

The DMN and the CEN have consistently shown to increase in their interconnectivity during both meditation (Brewer *et al.* 2011; Marzetti *et al.* 2014; Bauer *et al.* 2019) and the RS (Brewer *et al.* 2011; Marzetti *et al.* 2014; Creswell *et al.* 2016; Bauer *et al.* 2019; Kral *et al.* 2019) (Table 5). Although, it is evident that the relationship between the DMN and CEN is not always constant, with several studies showing that increased meditation proficiency/mindfulness is associated with increased negative connectivity between the two networks. This may be a result of a nonlinear trend (Bauer *et al.* 2019), much like that found for CEN activation (Brefczynski-Lewis *et al.* 2007), emphasizing that process of gaining meditation proficiency is not a linear path. Specifically, Bauer *et al.* (2019) found that meditators with an intermediate amount of experience (with respect to the full sample of participants) showed increasing anticorrelation between the DMN and CEN. Yet more advanced meditators showed a decrease of this anticorrelation—that is referred to being the “ultimate network reconfiguration” since it represents a configuration where the CEN no longer has to interact and interfere with the DMN’s spontaneous activity (Fig. 6). This leads highly experienced meditators brains to exhibit a positive connectivity between the DMN and CEN (Brewer *et al.* 2011). This coactivation of the DMN and CEN is a key finding in support of nondual awareness (Josipovic *et al.* 2012). This is not conclusive, although, and it must also be stated that different meditation strategies seem to impact the interaction between these networks in a distinct way (Josipovic *et al.* 2012). More research is highly encouraged to determine the relationship between meditation type, meditation proficiency, and DMN–CEN connectivity.

**Table 5.** Primary DMN–CEN connectivity findings associated with meditation

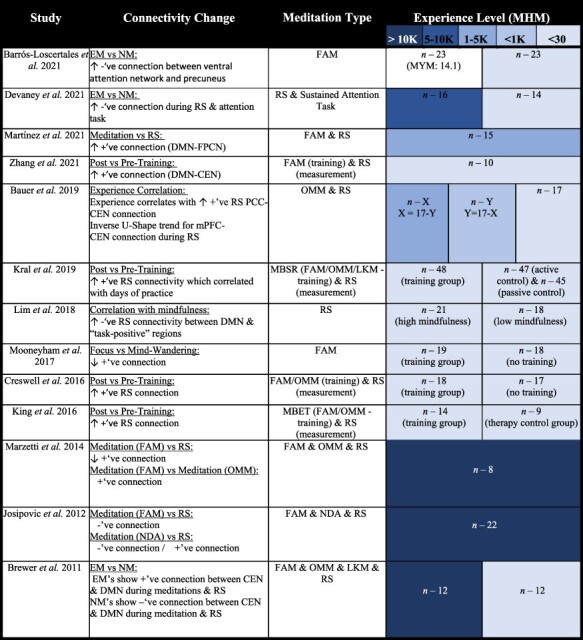

Supporting Information: Text within sections allocated to MHM is to specify either the differentiating factor between groups of similar experience level (e.g. meditation training vs relaxation training) or the level of meditation when mean hours of experience were not provided within the published paper.

Abbreviations: experienced meditators (EM); frontal-parietal control network (FPCN); medial prefrontal cortex (mPFC); mean hours of meditation (MHM); mindfulness-based experiential therapy centre (MBET); mindfulness-based stress reduction (MBSR); naïve meditators (NM); nondual awareness meditation (NDA).

#### SN as a potential mediator between the DMN & CEN

The SN, with anterior insula and dorsal anterior cingulate cortex (dACC) as key regions (besides other cortical and subcortical regions), plays a primary role in several distinct forms of meditation. One study that segregated a meditation session into four different states including “mind-wandering,” “awareness of mind-wandering,” “shifting attention,” and “sustained focus” found that both the anterior insula and the dACC were activated when the meditator became aware that there was no longer sustained attention toward the meditation object, in this case the breath (Hasenkamp and Barsalou 2012; Hasenkamp *et al.* 2012). This suggests that the SN may very well be a mediator between the DMN and the CEN (Spreng *et al*. 2010). This finding is in line with another study involving highly advanced Theravada Buddhist Monks that show an anticorrelation between the SN, i.e. dACC, and the CEN, i.e. middle frontal gyrus during focused meditation (Manna *et al.* 2010). Yet the relationship of the correlation between the SN and the CEN is not fully understood since other impactful studies have shown a coactivation of the SN and the CEN during several different types of meditation (Brewer *et al.* 2011; Kemmer *et al.* 2015; Mooneyham *et al.* 2017).

Regarding the SN’s relationship with the DMN, there is a similar trend of ambiguity in the findings, with some studies suggesting a decreased connectivity during meditation, especially focused meditation (Kemmer *et al.* 2015). Yet some findings demonstrate a positive connectivity during open forms of meditation (Kilpatrick *et al.* 2011; Marzetti *et al.* 2014) and other forms (Manna *et al.* 2010; Brewer *et al.* 2011; Hernández *et al.* 2017). Doll *et al.*’s (2015) propose that mindfulness is represented by an increase in PCC-insula anticorrelation. Yet other studies show the opposite trend, both during meditation and RS (Brewer *et al.* 2011; Hernández *et al.* 2017). Henceforth, the exact mechanism and role of the SN are still uncertain. Although it is possible that the reason behind these contradictory findings is to do with a nonlinear trend of SN activity and connectivity with respect to meditation proficiency. This is supported by the finding of an inverse U-shape trend of anterior insula activity with respect to meditation experience (Brefczynski-Lewis *et al.* 2007).

**Table 6.** Main findings of studies regarding meditation and self-related processing

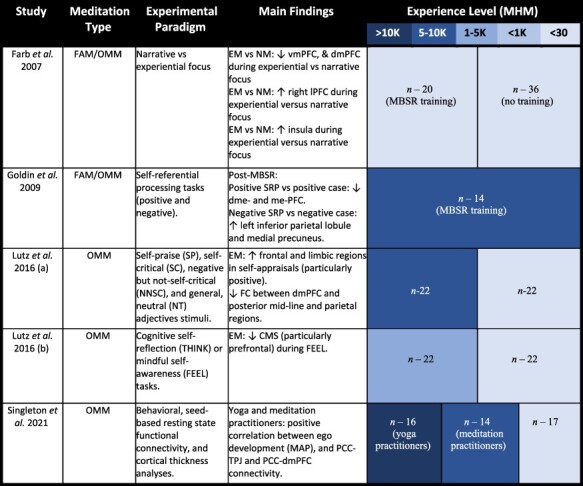

Supporting Information: Text within sections allocated to MHM is to specify either the differentiating factor between groups of similar experience level (e.g. meditation training vs relaxation training) or the level of meditation when mean hours of experience were not provided within the published paper.

Abbreviations: cortical midline structures (CMS); dorsal medial prefrontal cortex (dmPFC); experienced meditators (EM); focused awareness meditation (FAM); functional connectivity (FC); inferior frontal cortex (IFC); lateral prefrontal cortex (lPFC); naïve meditators (NM); mindfulness-based stress reduction (MBSR); precuneus (Pcu); self-referential processing (SRP); ventral medial prefrontal cortex (vmPFC).

### Interaction of meditation and self

We so far reviewed changes in DMN–CEN relationship as well as three layers of self during proficient meditation compared to beginning meditators. This suggests topographic reorganization of both CEN–DMN relationship and the three layers of self during proficient meditation. Does this mean that the mental self is diminished in the experienced meditators? In order to address this question, one would want to investigate the interaction of meditation × self within one study design. As there are only a few studies on that, we have included a summative table of each study and its respective findings and have included detailed descriptions of each in Table 6

Together, these findings confirm that experienced meditators exhibit a reorganization of the different layers of self-processing, that is instead misaligned in healthy dualistic experiences, as evident from the DMN–CEN anticorrelation. Hence, these findings confirm that experienced meditators show a less pronounced self-bias, which we suggest is linked to the DMN–CEN shift from an anticorrelation to a coactivation.

**Figure 7.**
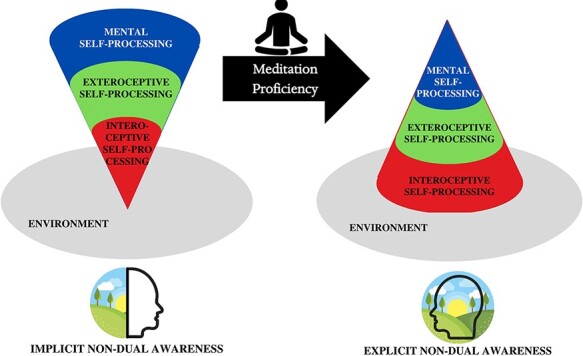
Synchronization between the three different layers of self-related processing due to increased meditation proficiency. The three layers of self-related processing are desynchronized in subjective states of implicit nondual awareness (i.e. duality) and impacted by hyperactive mental-self processing (likely tied with hyperactive PCC activity). Through increased meditation proficiency, the relationship between the layers of self-related processing likely becomes increasingly synchronized and nested within the environment, allowing for a present-moment orientation and a recognition of nondual awareness

## Discussion

We reviewed various imaging studies that focused on the impact that meditation proficiency, including its interaction with the self, has on spatial topography and connectivity features of the brain. In summary, the most consistent meditation-related findings of the review are (i) decreased posterior DMN (PCC) activity, (ii) increased CEN (dlPFC) activity, (iii) decreased connectivity within posterior DMN as well as between posterior and anterior DMN, (iv) increased connectivity within the anterior DMN and CEN, and (v) significantly impacted connectivity between the DMN and CEN (likely a nonlinear phenomenon) with a final stage of positive connectivity and coactivation. Additionally, the SN has been a common network showing meditation-related alteration in activity and connectivity and may serve as a mediator between the DMN and CEN (Hasenkamp and Barsalou 2012; Hasenkamp *et al.* 2012).

These results are in accordance with a topographic reorganization of the brain: this, as we postulate, leads to a nondual mode of neural functioning with decreased or nonexisting segregation between the DMN and CEN. We suppose that such nondual topographic organization is manifest on the psychological level in the dissolution of the self-other/environment duality into the experience of nondual awareness (Josipovic *et al.* 2012; Ataria *et al.* 2015; Josipovic 2019; Lindahl and Britton 2019). This inextricable tie between the neural topography and subjective phenomena associated with increased meditation proficiency is a primary aspect of the TRoM. The spatial organization or topography of the brain thus provides a link or “common currency” of objective (neural) and subjective (experiential) levels (see Northoff and Lamme 2020a; Northoff *et al.* 2020b).

We suggest that this topographic reorganization brings to the forefront the unveiling of a most basic, fundamental, experience-based level of self, which becomes explicitly aware of its own nondual nature, e.g. nondual awareness: the nondual organization of our mental life, at its very bottom, is characterized by strong self-body-environment alignment (see Josipovic 2021 for more on the transition from implicit to explicit nondual awareness). Not surprisingly, the region with the most consistent meditation-related finding throughout the reviewed studies (decreased PCC activity) has been reviewed in itself and was found to be associated with “getting caught up in” one’s experience, like getting caught in habits or viewpoints (Brewer *et al.* 2013). Proficient meditation allows the practitioner to release themselves from identification with and ownership of these habits and viewpoints, thus allowing them to be seen in the context of the overall present moment in which there is a nondual relationship between all phenomena, i.e. nondual awareness. We believe that this transformation, elicited through increased meditation proficiency, is dominantly characterized by a downshift in the dominance of mental self-processing and an increase of exteroceptive and interoceptive self-processing, hence leading to a new default state of the brain (Brewer *et al.* 2011). This increasingly expansive and integrated default mode has already been shown by Kajimura *et al.* (2020), where they found that the frontal-parietal network was increasingly merged with the DMN following meditation, leading them to suggest that aside from altered functional connectivity, “meditation leads to reconfiguration of whole-brain network architecture.”

### TRoM I: resetting the relationship of DMN and CEN

In subjective states in which nondual awareness is implicit, the duality on the neural level between specific regions/networks like the default-DMN as distinguished from the CEN (Northoff *et al.* 2006; van der Meer *et al.* 2010; Andrews-Hanna 2011; Anticevic *et al.* 2012; Andrews-Hanna *et al.* 2014; Murray *et al.* 2014; Davey *et al.* 2016; Frewen *et al.* 2020) is mirrored on the subjective duality between of self as distinguished from other (Wolff *et al.* 2018; Huang *et al.* 2016; Kolvoort et al. 2020; Northoff *et al.* 2020b). These findings suggest that the brain’s spatial topography with its duality of DMN and CEN is intimately related to the experienced duality of self and other/world. Accordingly, duality of self-other/world may be shared on neuronal and psychological levels as their “common currency” (Northoff and Huang 2017; Northoff and Lamme 2020a; Northoff *et al.* 2020b).

This changes during the practice of meditation and especially in the proficient stages. The topographic reorganization of the DMN–CEN relationship changes from primarily a negative to a positive correlation (see Fig. 6). This amounts to a topographic reorganization of the whole brain (see Kajimura *et al.* 2020 for a direct example of this). In the meditation-related transformed default mode of the brain, the initial DMN likely no longer stands in negative but in positive correlation to CEN, a network of the brain found earlier in the principal gradient system of the brain (Margulies *et al*. 2016). Due to this transformation, the default mode may now be spatially extended to and include non-DMN regions (see also Bauer *et al.* 2019 for a somewhat analogous suggestion of the brain’s transformed default mode). This means that, cognitively, both self and other/world-related regions, mediated through meditation experience, may simultaneously function in a new default manner: self- and other-related contents can now be processed and synchronized together, which, in turn, renders superfluous their distinction between the self and other/environment (Brewer *et al.* 2011; Hasenkamp *et al.* 2012; Marzetti *et al.* 2014; Bauer *et al.* 2019). Hence, we suggest that there is likely a correspondence between the dissolution of neuronal boundaries found between networks like the DMN and CEN and the dissolution of boundaries between the self and other on the psychological level of experience.

Although the state of explicit nondual awareness is one that is inherently effortless, it seems fair to say that effort is required in order to transform the correspondence between the DMN and CEN. Much like the mystic Osho has said in the context of Zen:

“Zen says, achieve it effortlessly; there is no need of effort. Zen is right; there is no need for an effort. But remember, to achieve this point of no-effort you will need a long, long effort.”

*Osho (2012)*

Similar to the taming of an elephant that entails binding an elephant to a large post in order to subdue its wild habits, it seems as although the mind must undergo a similar treatment through meditation, an idea already presented in early Buddhist accounts and the modern “Handbook of Mindfulness and Self-Regulation” (Ostafin 2015). The elephant, based on the most replicated fMRI-related finding on meditation, would be the posterior section of the DMN, notably the PCC, while the tamer would likely be that of the CEN, with the dlPFC likely playing a primary role. Through effort, the meditator begins to learn to subdue the hyperactivation of the PCC and cultivate an increasing level of integration between the functional networks of the brain.

It should be noted that the explicit experience of nondual awareness may be increasingly likely during a more open form of attention like OMM, LKM, or NDA, as opposed to FAM. Most, if not all meditation practices, although, have a shared emphasis in their meditation practice on disengaging from mind-wandering and, at the same time, attending to the present moment—this can be observed in both focused or open attentional practices. We emphasize that all forms of meditation are ultimately progressive steps along the continuum of awareness with the ultimate realization being that of nonduality. So, although distinct meditation forms have different neural correlates, we suppose that they all most likely induce a topographic DMN–CEN reorganization, which, in either case, is associated with the experience of nonduality (see Deepeshwar *et al.* 2019; Schoenberg and Vago 2019 for precise stage-wise progressions). Given that different forms of meditation may all ultimately lead to nonduality, this is very much like the analogy proposed by the 15th-century Zen Buddhist Monk, Ikkyu: “many paths lead from the foot of the mountain, but at the peak we all gaze at the single bright moon.”

### TRoM II: A transformed relationship of self-processing layers

Qin *et al.* (2020) propose a neural model subdividing self-processing into three intimately connected and spatially nested levels with different topographic extensions in the brain: interoceptive-processing, exteroceptive processing, and mental-self-processing, which is similar to previous models (Damasio 1999, 2003a, 2003b, 2010). Interoceptive self-processing refers to the brain’s interaction with the environment at a physiological level, that is, for example, through gastrointestinal and cardiorespiratory sensations (Craig 2009; Seth 2013; Tsakiris and Critchley 2016). At the second layer, the interaction with the environment at a proprioceptive/affective level is processed, that is for example, through what we refer to as bodily and emotional perceptions. At the third layer, it is mental/cognitive self-processing, which consists of representations of self-related nonbodily signals like personal traits and other mental contents that take place.

Recruiting a variety of different imaging findings on both meditation and self, we suppose that the three layers of self (interoceptive, exteroceptive, and mental/cognitive) become more strongly synchronized between each other, allowing a better alignment with the environment (see Fig. 7). This renders more explicit the otherwise more implicit connection of brain, body, and environment, which now becomes the focus of experience (see Glossary of Terms and Northoff and Zilio (2022) for a clarification of terminology).

It is important for us to underline that the partition of self-processing in the above-described three layers is only a conceptual subdivision, meaning that the mechanism of self-processing is better to be thought of as a continuum. Specifically, presupposing this hierarchical model of self (Qin *et al.* 2020), we suppose that the upper mental layer is featured by the dichotomy of self and nonself whereas that is not yet present on the more basic layers of self, especially, the self of the interoceptive and exteroceptive layers. As featured by intero-exteroceptive connection, the lowest layer of self is, by default, organized in an increasingly nondual manner. If, through topographic reorganization, the upper mental/cognitive layer of self is synchronized with its intero-exteroceptive layers, the mental/psychological duality of self and nonself is immersed and integrated within the more nondual brain–body–environment connection of the primordial and immediate layers of self (Thompson and Varela 2001; Northoff and Zilio 2022).

In common dualistic experiences, nondual awareness is indeed always implicit (Josipovic 2021) and thus serves as a predisposition or prerequisite for dualistic experiences, which are fundamentally linked to higher-order cognitive processing (see Fig. 1). This is reflected in the hierarchical structure of the three-layered model of self (Qin *et al.* 2020): the regions of the intero-exteroceptive layers of self are recapitulated within the upper layers of self, that is, in mental/cognitive layers of self (where they are joined by the recruitment of additional regions). The hierarchy among the three layers of self is thus nested such that the lower layer is present within the next upper layer and so forth.

Such nested hierarchy carries major implications for the structure of consciousness: even if the dual organization of self and nonself of the mental level is predominant and is thus explicit in our experience, there is still a more implicit experience of nonduality in the background of experience. This seems to lead to an almost paradoxical situation: while the mental/cognitive layer is characterized by strong beliefs, categorization, and projection of a duality between self and other/environment (Josipovic 2021), there remains nevertheless a tacit feeling or implicit experience of nondual connectedness and integration of self and world in the background of our experience (Fig. 1).

Once the upper mental and lower intero-exteroceptive layers of self-processing are synchronized and thus function according to their nested and inter-related nature, then a nonconceptual recognition of nondual awareness by nondual awareness itself is possible: an auto-recognition of reflexive awareness. See, for example, phenomenological reports from one highly proficient meditator in a state of, what we would call, explicit nondual awareness:

“I’m not there basically, just world, so there’s no real location at all (…). It’s very minimal, almost nothing… When there’s no boundary, there’s no personal point of view, it’s the world point of view, it’s like the world looking, not [me] looking, the world is looking.”

“I dissolve into the world and where you have the boundaries and the self as foreground and the world as background or as ground, here there isn’t a ground, there isn’t a foreground, in a way the background is everything, although not identical to the world, I’m not separate from background.”

*Ataria et al. (2015)*

We suppose that the transition from implicit to explicit nondual awareness takes place when the fundamentally nondual intero-exteroceptive layers shift into the foreground of experience, while the usually more dominating dual mental self at the foreground now becomes nested within these more spatially and temporally expansive layers of self. Hence, we suppose a shift of background and foreground in consciousness: the focus on the mental self shifts to allow the intero-exteroceptive self, the usual more implicit background, to shift into the foreground of our experience and is thereby rendered explicit.

## Limitations and recommendations

Some limitations to our review and model need to be considered. First, not all findings of meditative studies form a consensus; thus, these concepts should be taken as approximations that are based on some of the most replicated results. Second, a breadth of meditation types has been included without an in-depth model of how each type is related to one another. This means that each of the study findings is not necessarily in corroboration with each other, rather they may be highlighting specific aspects of the meditation type, which was involved in the experimental procedure. More work is needed to find specific correlates associated with distinct meditation types along distinct stages of meditation proficiency. Finally, a large level of interindividual differences among subjects within the studies along with the studies’ various experimental paradigms (i.e. length of meditation session, specific instructions for meditation, bodily orientation during meditation, and bandwidth of data acquisition) likely play a role in the heterogeneity within the findings and the field would benefit from controlling and directly assessing these variables in later research.

In order to verify and/or modulate the viewpoints developed here, there will need to be further direct testing, with the recommendation of involving experiential questionnaires in order to accurately link neural features to experiential phenomena. Another limitation has been the scarce amount of meditation literature that included in its experimental paradigm the distinction between the different layers of self-processing, i.e. mental-self-processing (or narrative self) and intero-exteroceptive processing (or minimal/experiential/core self). In our opinion, this means that although many studies have focused on the neural correlates of meditative states and traits and on self-dissolution experiences, little attention has been devoted to building studies that focus on how self-processing evolves with increased meditation proficiency and how this correlated with neural measures.

Finally, we encourage future meditation research to incorporate scales of mindfulness such as the Mindfulness Attention Awareness Scale (Brown and Ryan 2003) and nonduality (Hanley *et al.* 2018) in order to avoid the vagueness of meditation experience levels in terms of hours and years. We deem that this is a limitation of the field since the amount of progression and understanding that one gains through meditation is extremely variable between individuals. This is made evident by the fact that individuals without meditation experience vary in levels of mindfulness. Introducing these scales will allow for more comprehensive correlational analyses to take place, which can more accurately relate the changes in the brain to the proficiency level of the meditator.

## Conclusion

Our review illustrates that meditation has a profound neural reorganization on the brain’s spatial topography, particularly in the relation between the DMN and CEN. The shift from naïve to highly proficient meditation likely allows for a reorganization of the brain’s spatial features on the neural level, which leads to a transformed default mode in which the functional networks are increasingly synchronized and thus can operate in an increasingly cooperative manner. Based on preliminary evidence, we suggest that this may manifest on the psychological level as a transition from implicit to explicit nondual experience of self and other/world.

We therefore develop the TRoM that postulates the spatial-topographic (and, in the future, also temporal dynamic) reorganization of the brain from naïve to highly proficient stages of meditation. The key hypothesis of TRoM is that brain and henceforth the psyche (or mind) are organized in a nondual way: this emphasizes the nondual connection of self and nonself/environment on a deeper intero-exteroceptive level over their duality that predominates on the more superficial mental level. On a more general level, this lends further support to the assumption that neural and mental levels share most basic spatial-topographic (and temporal dynamic) features as postulated in the “common currency hypothesis” of brain and mind (Northoff and Lamme 2020a; Northoff *et al.* 2020b).

Proficient meditation unravels a deeper social and ecological intero-exteroceptive layer of self where it is intimately connected with the environment in a nondual manner. This hints upon a more basic and fundamental notion of self where it is determined by and through its relationship to the other/environment. Reaching beyond the duality of self-other/environment, this deeper relational layer of self is intrinsically neuro-ecological and neuro-social. That connects this deeper layer of self intimately with both other and world in a positive way, that is, integrated and rather than segregated. Proficient meditation can thus provide us with a deeper level in our experience of the own self, a nondual experience where our self is integrated with the other and part of the wider world, as discussed in previous neurophenomenological models of explicit nondual awareness (Josipovic 2019, 2021). This allows to lay bare a deeper ecological and social nondual layer not only of ourself but, correspondingly, also of our brain featured by its deeper most basic and fundamental nondual spatial topography serving as its default or baseline.

## Supplementary Material

niac013_Supp

## Data Availability

The technical details and main findings from all reviewed studies may be found in the Supplementary Material.

## Glossary of Terms

Definitions:

**Temporo-spatial nestedness and alignment:** this provides the necessary neural conditions of possible consciousness, that is, the neural capacity or neural predisposition and prerequisite of consciousness

**Temporo-spatial nestedness:** this refers to a particular type of organization of different spatial and temporal scales of

neural activity. Rather than operating in parallel, unconnected, or causally connected but separate ways, the different spatial and temporal scales of neural activity are contained or nested within each other.

**Temporo-spatial alignment:** this refers to the phenomenon in which the brain encodes the context by adapting (and thereby aligning) its own neural activity to the various inputs/stimuli that shape the context.

**Self-related processing:**

**Interoceptive self:** this represents internal sensory signals for cardiorespiratory, gastrointestinal, and urogenital functions. The prerequisite of integration of internal and external environmental signal into the self.

**Exteroceptive self:** this represents exteroceptive and proprioceptive signals directly relates to one’s own body. An intermediate level that links the internal body and external environment.

**Mental-self-processing:** this represents self-related nonbodily signals, such as personal traits and other mental contents and finalizes the integration of external environment to the self

**Nondual awareness:** this is a background awareness that precedes conceptualization and intention and that can contextualize various perceptual, affective, or cognitive contents without fragmenting the field of experience into habitual dualities (Josipovic 2013)

## Abbreviations

TRoM: topographic reorganization model of meditationDMN: default mode networkCEN: central executive networkmPFC: medial prefrontal cortexdlPFC: dorsal lateral prefrontal cortexPCC: posterior cingulate cortexACC: anterior cingulate cortexRS: resting state
