# Supplementary material for: Beyond the veil of duality—topographic reorganization model of meditation
Source: Neurosci Conscious. 2022 Sep 27;2022(1):niac013. doi: 10.1093/nc/niac013 (PMC9552929; doi:10.1093/nc/niac013)
Supplement: niac013_Supp [file niac013_supp.zip › TRoM - Supplementary Material - ReRe NoC.docx]

# Beyond the Veil of Duality – Topographic Reorganization Model of Meditation

Supplementary Material

Abbreviations:

ACC (Anterior Cingulate Cortex), AG (Angular Gyrus), CEN (Central Executive Network), CMS (Cortical Midline Structures), dACC (Dorsal Anterior Cingulate Cortex), DAN (Dorsal Attention Network), dlPFC (Dorsal Lateral Prefrontal Cortex), DMN (Default Mode Network), EM (Experienced Meditators), FAM (Focused Awareness Meditation), FEF (Frontal Eye Field), FPN (frontal parietal control network), IBMT (Integrative Body-Mind Training), IFC (Inferior Frontal Cortex), IPG (Inferior Parietal Gyri), IPL (Inferior Parietal Lobule), ITC (Inferolateral Temporal Cortex), LKM: (Loving Kindness Meditation, LOG (Lateral Orbital Gyrus), Mindfulness Based Experiential Therapy Centre (MBET), Mindfulness Based Stress Reduction (MBSR), MCG (Middle Cingulate Gyrus), meFG (Medial Frontal Gyrus), MFG (Middle Frontal Gyrus), MHM (Mean Hours of Meditation), mid cing. (Middle Cingulate), mPFC (Medial Prefrontal Cortex), MRM (Mantra Recitation Meditation), mSFG (Middle Superior Frontal Gyrus), MTC (Middle Temporal Cortex), MTG (Middle Temporal Gyrus), MYM (Mean Years of Meditation), Nondual Awareness Meditation (NDA), NM (Naïve Meditators), OMM (Open Monitoring Meditation), PCC (Posterior Cingulate Cortex), pgACC (Pregenual Anterior Cingulate Cortex), ReHo (Regional Homogeneity), RS (Resting State), rsFC (Resting State Functional Connectivity); SG (Subgroup), sgACC (Subgenual Anterior Cingulate Cortex), SN (Salience Network), SPL (Superior Parietal Lobule), vlPFC (Ventrolateral prefrontal Cortex), vmPFC (Ventral-medial Prefrontal Cortex), vOFC (Ventral Orbitofrontal Cortex),

## Table S1: Summary of Studies Meditation Groups & Imaging Modality

| **Study** | **Meditation Type** | **Number of Subjects** | | **Imaging Modality** |
| --- | --- | --- | --- | --- |
|  |  | **Advanced Meditators** | **Naïve Meditators (MHM < 30)** |  |
| Avvenuti et al., 2020 | 3-Month Transcendental Meditation Training (MRM) |  | N – 34 (SG1: N – 19 [Training Group]; SG2: N – 15 [Control Group]) | fMRI |
| Bærentsen et al., 2010 | Breath Focus Meditation (FAM) &  MRM | N – 31 (MYM – 11) |  | fMRI |
| Baron Short et al., 2010 | Breath Focus (FAM) | N – 13 (MYM – 11.6 ± 9.4)  (SG1: N – 8 [MYM < 10]; SG2: N – 5 [MYM > 10]) |  | fMRI |
| Barrós-Loscertales et al., 2021 | Experience: Sahaj Yoga (FAM)  Scanning: RS | N – 23 (MYM – 14.1 ± 6.1) | N – 23 | fMRI |
| Bauer et al., 2019 | Goenka Vipassana (FAM/OMM) | N - 16 (MHM -  1677 ± 367; [SG1 - MHM < 1130];  [SG2 - MHM  > 1130]) | N – 17 | fMRI |
| Berkovich-Ohana et al., 2015 | MRM |  | N – 23 | fMRI |
| Berkovich-Ohana et al., 2016 | Breath and Body Focus Meditation (FAM) | N – 18 (MYM – 16 ± 7.5; MYM – 9040) | N – 18 | fMRI |
| Brefczynski-Lewis et al., 2007 | Dot Concentration (FAM) | N - 14 (SG1: N – 4 [MHM – 19000];  SG2: N – 4 [MHM – 44000]) | N – 16 | fMRI |
| Brewer et al., 2011 | Choiceless Awareness (OMM)  Breath Concentration (FAM)  Loving-Kindness Meditation (LKM) | N – 12 (MHM -  10565 ± 5158) | N – 12 | fMRI |
| Cotier et al., 2017 | 8-Week Meditation (FAM/OMM/LKM) Training |  | N – 30 (SG1: N – 16 [Training Group]; SG2: N – 14 [Relaxation Training]) | fMRI |
| Creswell et al., 2016 | 3-Day Mindfulness Training (FAM/OMM) |  | N – 35 (SG1: N – 18 [Training Group]; SG2: N – 17 [Controls]) | fMRI |
| Davanger et al., 2010 | ACEM (MRM) | N – 23 (MYM – 23) |  | fMRI |
| Devaney et al., 2021 | Meditation Experience: Varied  Analysis: RS only | N – 16 (MYM – 7 ± 6; MHM – 8311 ± 11682) | N – 14 | fMRI |
| Dickenson et al., 2012 | Breath Concentration (FAM) during Active Task |  | N – 31 | fMRI |
| Dodich et al., 2018 | Sahaja Yoga (FAM) |  | N - 42 | fMRI |
| Doll et al., 2015 | Training: Breath Focus (FAM)  Scanning: RS |  | N – 26 | fMRI |
| Engen et al., 2017 | LKM | N – 17 (MHM – 40000 ± 12000; MYM – 30 ± 7) | N – 15 | fMRI |
| Engström et al., 2010 | MRM | N – 8 (MYM – 1.167) |  | fMRI |
| Engström & Söderfeldt, 2010 | LKM/MRM | N – 1 (MYM > 6.5) |  | fMRI |
| Escrichs et al., 2019 | Anapanasati/ Breath Focus (FAM) | N - 20 (MHM - 9526.9 ± 8619.8) | N - 20 | fMRI |
| Farb et al., 2007 | 8 -Week Mindfulness Based Stress Reduction Program (MBSR) |  | N - 56 (SG1: N – 20 [8-week intervention]; SG2: N – 36 [Controls]) | fMRI |
| Farb et al., 2012 | Training: 8-Week Mindfulness Based Stress Reduction Training (MBSR) |  | N – 36 (SG1: N – 20 [Training Group]; SG2: N – 16 [Controls]) | fMRI |
| Fingelkurts et al., 2016 | 4-Month Meditation Course  (type was unspecific) |  | N – 10 | EEG |
| Froeliger et al., 2012 | Mindfulness (OMM) | N – 7 (MD[per week]M - 7 for MYM - 5.7 ± 3.8) | N – 7 | fMRI |
| Fujino et al., 2018 | FAM & OMM | N – 17 (MHM – 920.6 ± 573.7 |  | MRI |
| Gard et al., 2014 | Vipassana/ Insight (FAM/OMM) | N - 16 (MHM - 7458 ± 5734) | N - 15 | fMRI |
|  | Kripalu Yoga | N - 16 (MHY - 13534 ± 9950) |  |  |
| Garrison et al., 2013a | Breath Focus Meditation (FAM) | 10 (MHM: 10567 ± 4276; MYM: 18.4 ± 4.9) |  | fMRI |
| Garrison et al., 2013b | Breath Focus Meditation (FAM) | N – 22 (MHM: 9249 ± 1449; MYM: 12.0 ± 1.6) | N – 22 | fMRI Neurofeedback |
|  | Intentional PCC Deactivation | N - 9 (MHM: 8803 ± 3282; MYM: 9.5 ± 2.4) | N – 11 |  |
|  | Breath Focus Meditation (FAM) | N - 10 (MHM: 10567 ± 4276; MYM: 18.4 ± 4.9) |  |  |
| Garrison et al., 2014 | LKM | N – 20 (MHM of FAM – 9675 ± 1586 & MHM of LKM – 752 ± 217) | N – 26 | fMRI |
| Garrison et al., 2015 | Choiceless Awareness (OMM)  Breath Concentration (FAM) | N – 20 (MHM – 9676 ± 1586) | N – 26 | fMRI |
| Guleria et al., 2013 | “SOHAM” Meditation (MRM) | N – 14 (MHM – 2088 ± 320) |  | fMRI |
| Harrison et al., 2018 | Trait Mindfulness |  | N – 36 | fMRI |
| Hasenkamp et al., 2012 (a & b) | Breath Concentration (FAM) | N – 14 (SG1: N – 9 [MHM < 1200]; SG2: N - 5 [2000 < MHM < 4000]) |  | fMRI |
| Hernández et al., 2015 | Sahaja Yoga (FAM)  Breath Focus (FAM)  Mindfulness (FAM/OMM) | N – 19 (MYM – 11.8 ± 7.4) |  | fMRI |
| Hernández et al., 2017 | Sahaja Yoga (FAM) | N – 23 (MYM – 14.1 ± 6.1) | N – 23 | fMRI |
| Hölzel et al., 2007 | Breath Focus Meditation (FAM) | N – 15 (MYM – 7.9 ± 5.1) | N – 15 | fMRI |
| Ives-Deliperi et al., 2011 | Mindfulness (OMM) | N – 10 (MYM > 4) |  | fMRI |
| Jang et al., 2011 & 2018 | Training: FAM  Scanning: RS | N – 35 (MYM – 3.3 ± 2.1) | N – 33 | fMRI |
| Jao et al., 2016 | Taoist Meditation (FAM/OMM) | N – 18 (MYM – 7.4 ± 6.9) |  | fMRI |
| Josipovic, 2012 | FAM & NDA | N – 22 (MHM – 13727 ± 8920 |  | fMRI |
| Kajimura et al., 2020 | Breath Focus Meditation (FAM) |  | N – 1 | fMRI |
| Kemmer et al., 2015 | Breath Concentration (FAM) | N – 12 (MY[of daily]M > 3) | N – 12 | fMRI |
| Kilpatrick et al., 2011 | 8-Week Mindfulness Based Stress Reduction (MBSR)Training Course |  | N – 32 (SG1: N – 17 [Training Group]; SG2: N – 15 [Controls]) | fMRI |
| King et al., 2016 | 16-week mindfulness based exposure therapy |  | N – 23 (SG1: N – 14 [Training Group]; SG2: N – 9 [Control Therapy -no mindfulness]) | fMRI |
| Kral et al., 2019 | 8-Week Mindfulness Based Stress Reduction (MBSR) Course |  | N – 140 (SG1: N – 48 [Training Group]; SG2: N – 47 [Active Controls]; SG3: N – 45 [Passive Controls]) | fMRI |
| Kwak et al., 2019 | 4-Day Meditation Training (Templestay Program [FAM/OMM]) |  | N – 67 (SG1: N – 44 [Training Group]; SG2: N – 23 [Controls] | fMRI |
| Kwak et al., 2020 | 4-Day Meditation Training (Templestay Program [FAM/OMM]) |  | N – 37 (SG1: N – 23 [Training Group]; SG2: N – 14 [Relaxation Group]) | fMRI |
| Lazar et al., 2000 | Kundalini Meditation (FAM/MRM) | N – 4 (MYM > 4) |  | fMRI |
| Lim et al., 2018 | Resting State (RS) |  | N – 39 (SG1: N – 18 [Low Mindfulness Trait]) SG2: N – 21 [High Mindfulness Trait]; | fMRI |
| Liou et al., 2005 | Chinese Original Quiet Sitting (FAM/OMM) | N – 3 (10-21 years of meditation) |  | fMRI |
| Lo et al., 2017 | Zen Meditation | N – 8 (MYM – 5-12) | N – 8 | EEG |
| Lutz et al., 2016 | Training: Vipassana (FAM/OMM)  Scanning: Self-Appraisal | N – 22 (MHM – 5971 [Range: 506-18805]) | N – 22 | fMRI |
| Mahone et al., 2018 | Transcendental Meditation (MRM) | N – 16 (MHM > 36,000) |  | fMRI |
| Manna et al., 2010 | Vipassana (OMM)  Samatha (FAM) | N – 8 (MHM -15750 ± 9900) | N – 8 | fMRI |
| Martínez et al., 2021 | Body Sensation Focus (FAM) | N – 15 (MHM: 1677 ± 367) |  | fMRI |
| Marusak et al., 2018 | Resting State Functional- Connectivity (RSFC) |  | N - 42 | fMRI |
| Marzetti et al., 2014 | Vipassana (OMM)  Samatha (FAM) | N – 8 (MHM -15750 ± 9900) |  | MEG (2-80 Hz) |
| Miyoshi et al., 2019 | Breath Counting (FAM) |  | N – 29 | fMRI |
| Mishra et al., 2017 | AUM Mantra (MRM) & Visualization | N – 4 (No Experience Level Available) |  | fMRI |
| Mishra et al., 2020 | Breath Focus Training (FAM) |  | N – 45 (SG1: N – 15 [Internal Attention Intervention]; SG2: N – 15 [Attention Based Games]; SG3: N – 15 [No Intervention]) | fMRI |
| Mooneyham et al., 2017 | Breath Counting (FAM) & 6-Week Cultivation of Mindfulness |  | N - 37 (SG1: N – 19 [Meditation training];  SG2: N – 18 [Controls]) | fMRI |
| Newberg et al., 2001 | EM - Tibetan Buddhism (FAM/OMM) | N – 8 (MYM > 15) |  | SPECT (CBF) |
| Newberg et al., 2010 | Experience: FAM | N – 12 (MYM > 15) | N – 14 | SPECT (CBF) |
| Pagnoni, 2012 | Breath Focus (FAM) | N – 12 (MYM > 3) | N - 12 | fMRI |
| Panda et al., 2016 | Raja Yoga (FAM) | N – 20 (MHM - 11332.5 ± 6009.86 | N – 20 | fMRI-EEG |
| Santarnecchi et al., 2021 | 8-Week MBSR Training (FAM/OMM) |  | N – 44 (SG1: N – 23 [Training Group]; SG2: N – 21 [Control Group]) | fMRI |
| Scheibner et al., 2017 | Breath Focus (FAM) & Sound Focus (FAM) |  | N – 20 | fMRI |
| Shao et al., 2016 | 8-Week Meditation Training (FAM/OMM/LKM) |  | N – 45 (SG1: N – 23 [Training Group]; SG2: N – 22 [Relaxation Training]) | fMRI |
| Simon et al., 2017 | Kundalini Yoga Course (Includes: Mantra Recitation [MRM]) |  | N – 8 | fMRI |
| Smith et al., 2020 | OMM/FAM Meditation App Training |  | N – 22 (SG1: N – 13 [Training Group]; SG2: N – 17 [Controls]) | fMRI |
| Tang et al., 2013 | 2-Week IBMT (FAM/OMM) |  | N – 60 (SG1: N – 33 [Training Group]; SG2: N – 27 [Relaxation Group]) | fMRI |
| Tang et al., 2015 | 5-Day IBMT (FAM/OMM) |  | N – 40 | SPECT (CBF) |
| Tang et al., 2017 | 2-Weeks IBMT (FAM/OMM) Training |  | N – 25 | fMRI |
| Taren et al., 2017 | 3-Day Mindfulness (FAM/OMM) Training |  | N – 35 (SG1: N – 18 [Training Group]; SG2: N – 17 [Relaxation Retreat]) | fMRI |
| Taylor et al., 2012 | Fixation on cross (FAM) | N – 13 (MHM - 6519 ± 14445) | N – 11 | fMRI |
| Telles et al., 2014 | FAM (transition from effort to effortless) | N – 26 (SG1: N – 16 [MHM – 288]; SG2: N – 10 [MHM – 6048]) |  | fMRI |
| Tomasino et al., 2016 | 8-Week Mindfulness Training (FAM/OMM) |  | N – 13 | fMRI |
| van Lutterveld et al., 2017 (a) | Effortless Awareness (OMM) w. Neurofeedback | N -16 (MHM – 6164 [Range: 1527-50978]) | N – 16 | EEG |
| Wang et al., 2010 | Mantra and Sequential Finger Touching or Mantra Aligned with Breathing (MRM/FAM) | N – 10 (MHM – 20000; MYM>30) |  | Perfusion fMRI (CBF) |
| Winter et al., 2020 | Content-Related Meditation (FAM) & Content-Free Awareness (NDA) | N – 1 (MHM > 50000) |  | fMRI & EEG |
| Xiao et al., 2019 | 8-Week Mindfulness Based Stress Reduction (MBSR) Course |  | N – 32 (SG1: N – 16 [Training Group]; SG2: N – 16 [Controls]) | fMRI |
| Xu et al., 2014 | ACEM Meditation (MRM) | N – 14 (MYM – 27 ± 9) |  | fMRI |
| Xue et al., 2011 | 1-Month IBMT (FAM/OMM) Training |  | N – 32 (SG1: N – 15 [Training Group]; SG2: N – 17 [Relaxation Training]) | fMRI |
| Yang et al., 2019 | 8-Week Mindfulness Training (FAM/OMM) |  | N – 14 | fMRI |
| Yang et al., 2016 | 8-Week Mindfulness Training (FAM/OMM) |  | NM – 13 | fMRI |
| Zeidan et al., 2013 | 4-Day Breath Focus Meditation (FAM) Training |  | N – 15 | fMRI (CBF) |
| Zhang et al., 2021 | 2-Month Meditation Training (FAM) |  | N – 10 | fMRI |
| Zheng et al., 2019 | Haptic Assisted Breathing Meditation (FAM)  Breath Counting (FAM) |  | N – 12 | Near Infrared Spectroscopy |

## Table S2: Network Activity Findings

| **Study** | **Network Activity** |
| --- | --- |
| Baron Short et al., 2010 | **CEN:**  **Meditation vs Colour Identification Task:** ↑ dlPFC activity  **SG2 vs SG1:** ↑ consistent activation of dlPFC  **SN:**  **Meditation vs Colour Identification Task:** ↑ ACC activity  **SG2 vs SG1:** ↑ consistent activation of ACC |
| Bærentsen et al., 2010 | **DMN:**  **Meditation vs RS:** ↓ dominantly in right hemisphere of precuneus, posterior cingulum, and parietal-temporal area at beginning of meditation  **General:**  **Meditation vs RS:** ↑ bilateral putamen & r supplementary-motor cortex at beginning of meditation  ↑ head of nucleus activity during sustained meditation  ↓ activity in white matter, especially right lateralized, in posterior-occipito-parieto-temporal area and frontal lobes |
| Bauer et al., 2019 | dlPFC, SPL, & TPJ activity positively correlated with mindfulness |
| Berkovich-Ohana et al., 2015 | **DMN:**  **Meditation vs RS:** ↓ of widespread activity during MRM, centralized in DMN |
| Brefczynski-Lewis et al., 2007 | **DMN:**  **EM (SG1 &SG2) vs. NM:** ↓activity in PCC/precuneus & meFG/ACC **SG1 vs. NM:** ↑ mid cing., ant. ins., thalamus **SG1 vs. SG2:** ↑ l IPS, mid. cing., ant. ins., thalamus  **CEN:**  **SG1 vs. NM:** ↑ FEF  **SG1 vs. SG2:** ↑dlPFC & FEF activation duration throughout FAM |
| Brewer et al., 2011 | **DMN:**  **EM vs NM:**  ↓ activity during FAM & LKM for mPFC & PCC  Equal activation during OMM, excluding S & MTG |
| Davanger et al., 2010 | **CEN:**  **Meditation vs Concentrative Cognitive Tasks:** **↑** IFG which did not habituate over time, rather it increased in strength with time meditating |
| Dickenson et al., 2012 | **DMN:**  **MW vs Meditation:** ↑ mPFC/rACC, dmPFC, PCC, PCu  **Meditation vs MW:** ↑ dmPFC, r TPJ  **SN:**  **Meditation vs MW:** ↑ r mid insula, r dACC |
| Doll et al., 2015 | **DMN:**  **Meditation vs RS:** ↑ dmPFC activation |
| Engen et al., 2017 | **CEN:**  **EM vs NM:** ↑ ALFF for *meditation vs. RS* comparison in l frontopolar cortex  **General:**  **EM vs NM:** ↓ ALFF for *meditation vs. RS* comparison in l centroparietal and l medial occipital regions |
| Engström et al., 2010 | **General:**  **Meditation vs RS:** ↑ activity in r hippocampus, r precentral gyrus, bilateral MCG, & bilateral precuneus gyrus  **Active Task (“Table” & “Chair” Recitation):** ↑ activity in bilateral STG & l SFG |
| Engström & Söderfeldt, 2010 | **General**  **Meditation vs RS:** ↑ activation in l mPFC extending to ACC, in r caudate body extending to r insula, in l midbrain close to hypothalamus, & in l postcentral gyrus |
| Farb et al., 2007 | **DMN:**  **Training Group:**  ↓ dmPFC, vmPFC, & l dorsal amygdala during OMM  **Control Group:**  No differences between OMM and RS  **CEN:**  **Training Group:**↑ dPFC, inferolateral PFC, insula during OMM  **SN:**  **Training Group:**↑ insula during OMM |
| Farb et al., 2012 | **DMN:**  **Trained vs Control group:** ↓ vmPFC, & dmPFC during experiential focus meditation compared to narrative focus  **CEN:**  **Trained vs Control group:** ↑ right lPFC during experiential focus meditation compared to narrative focus  **SN:**  **Trained vs Control group:** ↑ insula during experiential focus meditation compared to narrative focus |
| Garrison et al., 2013a | **DMN:**  **Undistracted Awareness Correlate:** ↓ PCC activity  **Distracted Awareness Correlate:** ↑ PCC activity |
| Garrison et al., 2013b | ***Experiment 1***  **DMN:**  **Focused Attention Correlate:** ↓ PCC activity  **Self-Referential Activity:** ↑ PCC activity  ***Experiment 2***  **DMN:**  **EM vs NM:** ↑ volitional control to decrease PCC activity (no correlation between this control and the MHM of EM’s)  ***Experiment 3***  **DMN:**  **Focused Attention Correlate:** ↓ PCC activity – even when meditators did not know the meaning of the neurofeedback |
| Garrison et al., 2014 | **DMN:**  **EM vs NM:** ↓ PCC/PCu |
| Garrison et al., 2015 | **DMN:**  **OMM & FAM vs. RS & Active Task:** ↓ MTG, & precuneus activity  EM showed more ↓ compared to NM  **Active Task vs. RS:** EM showed ↑ Precuneus activity compared to NM  **SN:**  **OMM & FAM vs. RS & Active Task:** ↓ ACC,  EM showed more ↓ compared to NM  **Active Task vs. RS:** EM showed ↑ ACC |
| Guleria et al., 2013 | **DMN:**  **Meditation vs RS:** ↑ l middle PFC & l PCu  **CEN:**  **Meditation vs RS:** ↑ l IFG |
| Hasenkamp et al., 2012 (a) | **DMN:**  **MW:** ↑ PCC & mPFC  **Shift from MW to Focus:** vmPFC activated. SG1 show sustained activation, however, SG2 show momentary activation which falls back to baseline.  **CEN:**  **Shift to Focus:** ↑ r dlPFC, vlPFC & r PPL  **Focus:** ↑ dlPFC  **SN:**  **Aware of MW:** ↑ anterior insula, & dACC |
| Hernández et al., 2015 | **CEN:**  **Meditation vs RS:** ↑ activity in r mFG/ r IFG  **SN:**  **Meditation vs RS:** ↑ activity in r ACC & bilateral insula  **General:**  **Meditation vs RS:** ↑ activity in l & r superior/middle temporal region |
| Hölzel et al., 2007 | **DMN:**  **EM vs NM:** ↑ activity in dmPFC for meditation vs mental arithmetic comparison  **SN:**  **EM vs NM:** ↑ activity in rACC for meditation vs mental arithmetic comparison |
| Ives-Deliperi et al., 2011 | **DMN:**  **Meditation vs Visual Task:** ↓ mPFC & PCu  **SN:**  **Meditation vs Visual Task:** ↓ in insula & l vACC |
| Kwak et al., 2020 | **CEN:**  **Meditation-Training vs Control Training:** ↑ dlPFC activity during attentional task following meditation training compared to relaxation training  **SN:**  **Meditation-Training vs Control Training:** ↑ ACC activity during attentional task following meditation training compared to relaxation training |
| Lazar et al., 2000 | **SN:**  **Meditation vs Mental Animal Name Listing:** ↑ ACC  **CEN:**  **End of Meditation Session vs Beginning:** ↑ SFG, MFG, meFG  **General:**  **Meditation vs Mental Animal Name Listing:** ↑ putamen, midbrain, pregenual, & hippocampal/parahippocampal formation  **End of Meditation Session vs Beginning:** ↑ parietal lobule, SPL, IPL, STG, MTG, parahipocampal gyrus, precentral gyrus, postcentral gyrus, paracentral gyrus |
| Liou et al., 2005 | **General:**  **Meditation vs RS:** ↑ activation in pinear gland and hypothalamus during first & second stages of meditation |
| Lo et al., 2017 | **SN:**  Deep meditation showed inactive P3 & P4 channels |
| Lutz et al., 2016 | **DMN:**  ↓ in CMS (greater deactivation in prefrontal CMS for EM)  ↓ in amygdala for EM  **SN:**  ↓ in somatosensory activation |
| Mahone et al., 2018 | **CEN:**  **Meditation vs RS:** ↑ dlPFC  **SN:**  **Meditation vs RS:** ↑ ACC |
| Manna et al., 2010 | **DMN & SN:**  **EM - OMM vs. RS**: ↑ in l meFG, l SPL/precuneus, l STG activity Very similar to RS. ↑ in l ant. ins. compared to FAM  **EM - FAM vs. RS:** ↓ predominantly left lateral prefrontal, left anterior & posterior insula activity ↑ predominantly right medial frontal activity  **NM - OMM vs. RS:** ↑ l dACC, r rACC, r meFG, r IFG activity + correlation between l dACC & r medial aPFC  **NM - FAM vs. RS:** ↓ PCC activity |
| Mishra et al., 2017 | **CEN:**  **Meditation vs Relaxation:** ↑ r prefrontal regions activation in all participants |
| Newberg et al., 2010 | **EM vs NM:** ↑ CBF in prefrontal cortex, parietal cortex, thalamus, putamen, caudate, and midbrain |
| Newberg et al., 2001 | **Meditation vs RS:** ↑ CBF in CG, inferior FC, orbital FC, dlPFC, & thalamus |
| Pagnoni, 2012 | **DMN:**  **EM vs NM:** ↑ skewness in ventral posteromedial cortex, suggesting less states associated with elevated ventral posteromedial cortex activity. |
| Santarnecchi et al., 2021 | **Post vs Pre-Training** ↓ excitatory activity from bilateral ACC towards r anterior putamen when comparing post-training RS to pre-training RS  **Post vs Pre-Training** ↓ inhibitory activity from r cerebellum to post putamen when comparing post-training meditation to pre-training meditation  ↓ excitatory activity from l posterior putamen towards r cerebellum when comparing post-training meditation to pre-training meditation  ↑ inhibitory activity from l somatosensory cortex towards the r cerebellum when comparing post-training meditation to pre-training meditation |
| Scheibner et al., 2017 | **DMN:**  **Meditation vs MW:** ↓ in PCC, mPFC, & left temporal-parietal junction |
| Simon et al., 2017 | **DMN:**  **Meditation vs Finger Tapping:** ↓precuneus  Trend for ↓ of PCC  SN:  **Meditation vs Finger Tapping:** ↓ of pregeneal ACC |
| Tang et al., 2013 | **SN/DMN:**  **Training vs Control:** ↑ ACC/mPFC activity during RS  **CEN:**  **Training vs Control:** ↑ IFG/vlPFC activity during RS  **DMN:**  **Training vs Control:** ↓ precuneus/PCC activity during RS |
| Tang et al., 2015 | **Post vs Pre-Training:** ↑ CBF in vACC, mPFC, & insula |
| Telles et al., 2014 | **CEN:**  **One-Pointed Effortless Attention:** SG2 show ↑ in r MTC, r IFC, & l LOG |
| Tomasino et al., 2016 | **DMN:**  **Post vs Pre-Training:** ↓ rostralPFC & r parietal area  **CEN:**  **Post vs Pre-Training:** ↑ r dlPFC  **SN:**  **Post vs Pre-Training:** ↑ l caudate/anterior insula  ↓ r parietal area 3b |
| van Lutterveld et al., 2017 (a) | **DMN:**  **Effortless Awareness:** ↓ PCC activity (40-57 Hz) |
| Wang et al., 2010 | **General:**  **Depth of Meditation Correlation:** ↑ activity in l inferior forebrain (including insula, inferior frontal cortex, and temporal lobe) correlates with depth of meditation  **Meditation vs Meditation:** distinct activation patterns found between two meditation types. |
| Xiao et al., 2019 | **DMN:**  **Anxiety Correlate:**  ↑ PCC shows more anxiety  ↑ vmPFC show less anxiety  **SN:**  **Anxiety Correlate:**  ↑ ACC, anterior insula show less anxiety |
| Xu et al., 2014 | **DMN:**  **Nondirective MRM vs RS:** ↑ vmPFC, dmPFC, PCC/retrosplenial cortex, IPL, lateral temporal cortex, & hippocampal formation  **Nondirective MRM vs Concentrative MRM:** ↑ right medial temporal lobe (parahippocampal gyrus and amygdala) |
| Yang et al., 2019 | **DMN:**  **Post vs Pre-Training:** ↓ ALFF in precuneus/PCC during RS |
| Zeidan et al, 2013 | **SN:**  **Decreased Anxiety Related to Training: ↑** ACC & ant. Insula  **DMN:**  **Decreased Anxiety Related to Training:** **↑** vmPFC  **Increased Anxiety:** **↑** PCC |
| Zheng et al., 2019 | **General:**  **Meditation (finger-pressure meditation) vs RS:** ↑ uniform activation in prefrontal & sensorimotor regions  **Post-Meditation Training:** ↑ r prefrontal activation  ↑ FC between mPFC & sensorimotor cortex at RS |

## Table S3: Studies with Both Intra- and Inter-Network Connectivity Findings

| **Study** | **Intra-Network Connectivity** | **Inter-Network Connectivity** |
| --- | --- | --- |
| Avvenuti et al., 2020 | **DMN:**  **Training vs Control:** ↑ rsFC between PCC and both precuneus and l SPL (correlates with decreased anxiety) | **DMN-SN:**  **Training vs Control:** ↑ rsFC between PCC and r insula |
| Bauer et al., 2019 | **DMN:**  **EM vs NM:** EM had ↑ in precuneus and r AG connection | **DMN-CEN:**  **postOMM-RS vs preOMM-RS:** Experience correlates with ↑ PCC-CEN connection  **SG1 vs NM:** ↑ in anticorrelation between mPFC & CEN during RS **SG2 vs SG1:** ↓ in anticorrelation between mPFC & CEN during RS **SG2 vs NM:** No difference in anticorrelation between mPFC & CEN during RS |
| Berkovich-Ohana et al., 2016 | **DMN:**  **EM vs NM:** ↓ FC in DMN  **Meditation vs RS:** ↓ FC in DMN  **Meditation Experience Correlation:** -‘ve correlation between meditation experience and intra-DMN connectivity  **General:**  **EM vs NM:** ↓ FC in visual network  **Meditation vs RS:** ↓ FC in visual network | **DMN-Visual Network:**  **EM vs NM:** ↑ FC between DMN & visual network  **Meditation vs RS:** ↓ FC between DMN & visual network |
| Cotier et al., 2017 | **DMN:**  **Post vs Pre-Training:** ↓ FC in DMN for meditation vs relaxation training  ↓ nodal connectivity in PCC  **SN:**  **Post vs Pre-Training:** ↓ FC in SN for meditation vs relaxation training  **General:**  **Post vs Pre-Training:** ↓ FC in somatomotor network for meditation vs relaxation training  ↓ nodal connectivity in bilateral paracentral lobule & MCG | **DMN – Several Networks:**  **Post vs Pre-Training:** ↓ FC between DMN & the following: SN, visual network, & somatomotor network for meditation training whilst relaxation training showed opposite effect  **SN – Several Networks:**  **Post vs Pre-Training:** ↓ FC between SN & the following: somatomotor network & frontoparietal network for meditation training whilst relaxation training showed opposite effect |
| Doll et al., 2015 | **DMN:**  **Correlates of mindfulness:** ↓ between posterior and anterior DMN connectivity | **Correlates of mindfulness:** ↑ in PCC-insula anticorrelation |
| Froeliger et al., 2012 | **CEN:**  **EM vs. NM:** ↑ RSFC between r anterior IPS and l FEF, r MT and l FEF, r posterior and l anterior IPS and l MT | **DMN-CEN-SN:**  **EM (OMM vs. RS):** ↑ FC between FEF, medial dorsal thalamus, l lateral parietal, post. cerebellum, r posterior IPS, anterior IPS, r middle temporal  ↓ FC between l MT, dorsal mPFC, & dorsal insula  **Experience Correlates:** Experience shows greater (+)'ve RS correlation between l posterior IPS and mPFC, r anterior PFC & SN & weakening of RS connectivity between r posterior IPS and bilateral insula, and l MT and r anterior PFC |
| Garrison et al., 2014 | **DMN:**  **EM vs NM:** ↓ connectivity between PCC/PCu and other CMS | **CEN**  **EM vs NM:** ↑ PCC/PCu connectivity with l IFG & MFG  **SN**  **EM vs NM:** ↑ PCC/PCu connectivity with insula lobe |
| Harrison et al., 2018 | **DMN:**  **Trait Mindfulness Correlation:** ↓ intra-DMN rsFC | **DMN-Somatosensory Network:**  **Trait Mindfulness Correlation:** ↑ rsFC between DMN & somatosensory cortices |
| Hasenkamp et al., 2012 (b) | **DMN:**  **SG1 vs. SG2:** vmPFC less connected with PCC during FAM  **CEN:**  **Experience Correlation:** ↑ bilateral connection of dlPFC | **DMN-CEN-SN:**  **SG1 vs. SG2:** Less DMN connection with l OFC & vmPFC during FAM  **Focus Correlates:** ↑ connection between dlPFC & r insula  **Experience Correlation**  Correlated with decoupling of ACC from vmPFC & PCC Correlated with PCC, ACC, and vmPFC coupling with the vOFC |
| Jao et al., 2016 | **General:**  **Meditation vs RS:**  Areas with high degrees during RS show low degrees during meditation (including the sensory cortex)  ↑ degree in bilateral mPFC, bilateral dlPFC, bilateral insula, bilateral parietal cortex, bilateral PCC, bilateral precuneus, bilateral thalamus | **Meditation vs RS:**  ↑ long distance (anterior-posterior) edges & ↓ orthogonal (left-right) edges connecting bilaterally homologous cortices  ↑ Bilateral thalamus-PCC degree |
| Kemmer et al., 2015 | **DMN:**  ↓ in DMN within-module positive connectivity  **CEN:**  ↑ within-module positive connectivity in the CEN | **DMN-CEN-SN:**  **EM vs NM:** ↑ +’ve connection between frontoparietal attentional  circuits (CEN) & the anterior cingulate-insula-caudate network (SN) during FAM  ↓ in +’ve connection between DMN and SN during FAM  ↑ in -’ve connection strength between DMN & early visual areas during FAM |
| Marzetti et al., 2014 | **DMN:**  Negative correlation between MHM & PCC-l mPFC alpha connectivity | **DMN-CEN-SN:**  **FAM vs RS:**  ↓ in PCC alpha connectivity with l SFG, l SMFG, l LTC, & ACC  **OMM vs RS:** ↓ in PCC alpha connectivity with l IPS **OMM vs FAM:** ↑ in PCC alpha connection with l mPFC, l SFG, l ACC, l dlPFC, & l IPL |
| Taylor et al., 2012 | **DMN:**  **EM vs. NM:**  ↓ vmPFC connection with dmPFC and r ITC Weaker FC between DMN regions | **MG vs. NM:** ↑ r IPL connection with dmPFC, PCC & l IPL |
| Winter et al., 2020 | **DMN:**  **“Content-free-awareness” correlates:** ↓ FC in posterior DMN  **CEN:**  **“Content-free-awareness” correlates:** ↑ FC in DAN | **“Content-free-awareness” correlates:**  ↓ connectivity between DMN and primary auditory cortex (SN)  Unchanged connectivity between DMN & CEN |
| Xiao et al., 2019 | **DMN:**  **Meditation Training:** ↓ local synchrony in PCC | **Meditation Training:** ↑ FC between insula & postcentral gyrus  ↓ FC between precuneus and frontal medial gyrus |
| Yang et al., 2016 | **SN:**  **Post vs Pre-Training:** ↑ ReHo in dACC  **DMN:**  **Post vs Pre-Training:** ↑ ReHo in mPFC & TPJ during meditation  **General:**  **Post vs Pre-Training:** ↑ ReHo in l putamen during meditation | **General:**  **Meditation vs RS:** ↓ pgACC connectivity with bilateral IPG  ↓ dACC connectivity with l ant. insula  ↑ pgACC connectivity with mPFC, l STG, & r TPJ  **Post vs Pre-Training:** ↓ pgACC rsFC with precuneus/PCC, l dmPFC, r STG, l middle occipital gyrus, l ITG  ↑ pgACC rsFC with r ITG, rIFG, r TPJ/IPL  ↓ dACC rsFC with calcarine sulcus & cuneus  ↑ dACC rsFC with cerebellum, r IPL, & PCC |

## Table S4: Studies with Intra-Network Connectivity Findings

| **Study** | **Intra-Network Connectivity** |
| --- | --- |
| Barrós-Loscertales et al., 2021 | **DMN:**  **EM vs NM:** ↓ rsFC between bilateral precuneus/cuneus cortices & r AG  **CEN:**  **EM vs NM:** ↑ rsFC between l vlPFC & r dlPFC  **SN:**  **EM vs NM:** ↓ rsFC between l insula & midcingulate cortex |
| Fingelkurts et al., 2016 | **DMN:**  ↑ operational synchrony of anterior DMN  ↓ operational synchrony of posterior DMN |
| Fujino et al., 2018 | **DMN:**  ↓ connection between striatum and PCC |
| Jang et al., 2011 | **DMN:**  **EM vs NM:** ↑ connectivity in anterior DMN (mPFC) |
| Lo et al., 2017 | **CEN:**  ↑ in coactivation of Fz, FCz, & Cz in deep meditation |
| Mishra et al., 2020 | **SN:**  **Meditation Training vs Other Groups:** ↑ rsFC of dACC in cingulo-opercular network  **Correlation:** ↑ rsFC of dACC in cingulo-opercular network correlates with improvements in sustained attention, hyperactivity, and academic performance |
| Panda et al., 2016 | **DMN:**  **EM vs NM:** ↓ in PCC connectivity  ↑ frontal and temporal connectivity of DMN  **CEN:**  **EM vs NM:** ↑ in right frontal & left temporal connectivity |
| Santarnecchi et al., 2021 | **SN:**  **SG1 vs SG2:** ↓ RS ReHo within r insula  **CEN:**  **Post vs Pre-Training** ↓ ReHo within r MFG when comparing post-training meditation to pre-training RS  **General:**  **SG1 vs SG2:** ↓ RS ReHo within r anterior putamen & r cerebellar lobule  **Post vs Pre-Training** ↓ ReHo within l IPL when comparing post-training RS to post-training meditation |
| Taren et al., 2017 | **CEN:**  **Training vs Control:** ↑ rsFC between l dlPFC & r IFG, r MFG, r supplementary eye field, r parietal cortex, & l MTG |
| Xue et al., 2011 | **SN:**  **Post vs Pre-Training:** ↑ nodal efficiency/degree in l ACC for IBMT compared to relaxation  **General:**  **Post vs Pre-Training:**  ↓ nodal efficiency/degree in r supplementary motor area |

## Table S5: Studies with Inter-Network Connectivity Findings

| **Study** | **Inter-Network Connectivity** |
| --- | --- |
| Brewer et al., 2011 | **DMN-CEN-SN:**  **EM vs NM:**  +’ve connectivity vs –‘ve connectivity for PCC-dACC & PCC-dlPFC, during RS, FAM, OMM, & LKM  ↑ +’ve connection between mPFC & FG, ITG, parahippocampal gyri, & left posterior insula |
| Creswell et al., 2016 | **DMN-CEN:**  **Post vs Pre-Training:** ↑ RS PCC-dlPFC FC |
| Devaney et al., 2021 | **DMN-CEN:**  **EM vs NM:** ↑ anticorrelation between DMN & DAN during RS & sustained attention task |
| Doll et al., 2015 | **Meditation vs RS:** ↑ l PFC-amygdala correlates with mindfulness (MAAS) |
| Farb et al., 2007 | **DMN-SN:**  **EM vs NM:**  ↓ insula-vmPFC connection is uncorrelated for EM, but significantly coupled for NM |
| Hernández et al., 2017 | **DMN-SN:**  **“Mental Silence” Correlate:** ↑ FC between rACC/mPFC & anterior insula/putamen (observed only in meditation)  ↓ FC between rACC/mPFC & r thalamus/parahippocampal gyrus (observed in both meditation and RS) |
| Kajimura et al., 2020 | **DMN-CEN:**  **Longitudinal Network Transformation through Meditation:** ROI’s shifted from the frontal-parietal control network (encapsulated in our terminology of CEN) to the DMN, though this shift rerouted back to baseline at later stages of meditation training.   - CEN showed enhanced flexibility under meditation condition |
| Kilpatrick et al., 2011 | **DMN-SN:**  **Trained vs Untrained (during OMM):**  ↑ dmPFC-dACC connectivity (“positive coherence”)  ↑ sACC-visual cortex anticorrelation  ↑ connection between auditory cortex and the dmPFC  ↑ anticorrelation between sACC and visual cortex |
| King et al., 2016 | **DMN-SN-CEN:**  **Post vs Pre-Training:** mindfulness based therapy showed increased rsFC between PCC and both dlPFC & dACC, compared to the non-mindfulness based therapy  **Correlation:** ↑ PCC-dlPFC connectivity correlated with improvement in PTSD avoidant and hyperarousal symptoms |
| Korponay et al., 2019 | **General:**  **EM vs NM:** ↑ +’ve rsFC between wide range of areas (seed regions showing ↑ rsFC with global regions are: substantia nigra, ventral rostral putamen, inferior ventral striatum, ventral tegmental area) |
| Kral et al., 2019 | **DMN-CEN:**  **RS FC Post vs. Pre-Intervention:**↑ PCC-dlPFC FC post-intervention  PCC-dlPFC +’ly correlated with days of practice |
| Kwak et al., 2019 | **SN-DMN:**  **Post vs Pre-Training:** ↑ l rACC RS FC with dmPFC, PCu, & AG |
| Jang et al., 2018 | **EM vs NM:** ↑ insula FC with thalamus, caudate, MFG, & STG  ↓ insula FC with parahippocampal gyrus |
| Josipovic, 2012 | **DMN-CEN:**  **FAM vs RS:**  ↑ anticorrelation between extrinsic and intrinsic systems  **NDA vs RS:**  ↓ in anticorrelation between extrinsic and intrinsic systems |
| Lim et al., 2018 | **DMN-CEN:**  **SG2 vs. SG1:** ↑ functional-state transitions during RS  ↑ time in "task-ready" state during RS, characterized by high within-network connectivity and high anticorrelation between task-positive regions and the DMN.  ↓ time in "idling" state during RS, characterized by low within-network connectivity and low anticorrelation between task-positive regions and the DMN |
| Manna et al., 2010 | **CEN-SN:**  **EM - FAM vs. RS:**  dACC & l MFG play teeter-totter role in maintaining focus  dACC & meFG have positive correlation  **EM – OMM vs RS:**  dACC & meFG have positive correlation  **NM – OMM vs RS:**  dACC & meFG have positive correlation  rACC & r MFG have positive correlation |
| Martínez et al., 2021 | **General:**  **Pre-Meditation:** ↑ time spent in state of low FC between DMN & FPN & DAN  **Meditation:** ↓ time spent in state of low FC between DMN & CEN  ↑ time spent in state where r FPN network interacts with DMN, l FPN interacts with DAN, and l & r FPN are decoupled  ↑ time spent in state where there is a strong connectivity between DMN & FPN with a segregation of DAN  **Post-Meditation:**  ↑ trend for less time spent in state of low FC between DMN & FPN & DAN |
| Miyoshi et al., 2019 | **Meditation vs RS:** ↑ in clustering coefficient in 8 nodes (l frontal Inferior operculum, r inferior occipital, r parahippocampal, r cerebellum, r middle cingulum, l cerebellum crus1, l inferior occipital, and r paracentral lobule) |
| Mooneyham et al., 2017 | **DMN-CEN-SN:**  **Breath Focus Task vs RS:**  ↑ CEN-SN connectivity (+’ly correlated with mindfulness)  ↓ CEN-DMN (mPFC) connectivity has +’ve correlation with mindfulness  ↑ DMN-CEN-SN connectivity has -‘ve correlation with mindfulness |
| Shao et al., 2016 | **General:**  **Post vs Pre-Training:** ↑ +’ve rsFC between PCC/PCu and pons compared to relaxation training. |
| Smith et al., 2020 | **DMN-CEN-SN:**  **Training vs Control:** **↑** rsFC between FPCN & l & r nucleus accumbens.  **↑** rsFC between DMN & SN  **Training time Correlation:** **↑** rsFC between lateral parietal cortex & supramarginal gyrus |
| Tang et al., 2017 | **General:**  **Post vs Pre-Training:** ↑ +’ve FC primarily between bilateral superior/middle occipital gyrus, bilateral frontal operculum, bilateral superior temporal gyrus, right superior temporal pole, bilateral insula, caudate and cerebellum |
| Zhang et al., 2021 | **DMN-CEN:**  **Post vs Pre-Training:** ↑ rsFC between PCC & DAN  **General:**  **Post vs Pre-Training:** ↑ rsFC between DMN & r middle temporal region, bilateral SPL & DMN, & bilateral SPL & DAN  **Meditation Experience Correlation:** rsFC between l SPL & mPFC is +’ly correlated with meditation experience |

## Interaction of Meditation and Self – Detailed Study Descriptions

In Farb and colleagues (2007), participants were subdivided in a pre-training waitlist group and a post mindfulness training (MT) group and instructed to assume distinct modes of self-focus. In particular, participants were asked, in response to reading trait-related adjectives, to engage either a ‘narrative’ focus, in which they had to think about what the adjective meant personally to them, or an ‘experiential’ focus, in which they had to monitor their moment-to-moment experience. During experiential focus, focal reductions in self-referential cortical midline regions (mPFC and in particular PCC), were observed in novices. In the MT group, experiential focus resulted in more pronounced reductions in the mPFC, along with an increased engagement of a right lateralized network, including the lateral PFC and viscerosomatic areas such as the insula, secondary somatosensory cortex and inferior parietal lobule. A strong coupling between the right insula and the mPFC in novices that was uncoupled in the MT group was also shown through functional connectivity analyses.

In Goldin and colleagues (2009) the effects of mindfulness-based stress reduction (MBSR) on self-referential processing were indagated with patients with social anxiety disorder (SAD). The authors used a self-referential processing (SRP) task in which the stimuli (25 positive and 25 negative social trait adjectives) were presented three times, once in each of three conditions, that were: self-referential (self-descriptive or not), valence identification (positive or negative affective meaning) and case identification (upper- or lowercase) - the last two being control conditions. Participants had to press one of two buttons to indicate whether a word was or was not self-descriptive, was positively or negatively valenced, or was in upper- or lowercase letters. They examined changes from pre- to post-MBSR for the contrast of positive SRP versus positive case and of negative SRP versus negative case. For positive SRP, there was evidence of decreased BOLD responses in brain regions related to self-processing (dorsomedial and medial PFC) and language processing (left inferior frontal gyrus). Negative SRP versus negative case processing resulted in increased brain responses in visual attention (left inferior parietal lobule and medial precuneus).

Lutz and colleagues (2016 a) investigated neural and behavioral correlates of self-criticism and self-praise in mid-to-long-term mindfulness meditators (LTM) compared to matched meditation-naïve participants (MNP). Participants were presented with blocks of individually selected positive (self-praise, SP), negative (self-critical, SC), negative but not-self-critical (NNSC), and general, neutral (NT) adjectives, and reported their affective state after the blocks. On the neural level, both SP and SC yielded more activation in the dmPFC in LTM compared to MNP, along with decreased functional connectivity to posterior midline and parietal regions in LTM compared to MNP during both self-related appraisals.

Lutz and colleagues (2016 b) also investigated experienced mindfulness meditators (LTM) and matched meditation-naïve participants (MNP) during mindful self-awareness (FEEL), the instructions being “feel into yourself, simply be aware of body sensations and/or emotions in this moment without trying to change them” and self-referential thinking (THINK), the instructions being “think about yourself, reflect who you are, what you do, like, etc.”, interspaced by blocks of REST. The authors reported somatosensory activations and decreases in CMS during FEEL for both groups, but significantly stronger decreases in prefrontal CMS in LTM. LTM further showed decreases in language-related and amygdala regions, but the latter was not significantly different between groups.

Furthermore, Singleton and colleagues (2021) investigated the neural correlates of maturity development, i.e., the development of the self throughout the lifespan (they referred to Jane Loevinger’s theory of adult development, termed ego development (1966)) in long-term meditators, long-term yoga practitioners, and demographically matched controls. Their results indicated that the correlation between ego development, measured by the Maturity Assessment Profile (MAP), and PCC-TPJ and PCC-dmPFC connectivity was positive for long-term meditators and long-term yoga practitioners, but negative for control participants.

Savostyanov et al. (2020), examining the effect of long-term meditation practice during an error-recognition task, presented emotionally negative (evoking anxiety or aggression) written sentences describing self-related or non-self-related emotional states and personality traits. They showed that, while naïve meditators perform better in tasks about self than about non-self, there is no such difference in experienced meditators. In addition, Katyal and colleagues (2020) examined pleasant and unpleasant self-views in long-term meditators versus age-matched meditation-naïve control participants. A self-referential encoding task (SRET) in which they presented 60 trials with 30 pleasant and 30 unpleasant trait adjectives taken from the Affective Norms for English Words (ANEW; Bradley & Lang, 1999) was used. After every trial, participants were required to answer to the question “does this word describe you?” by pressing the left or right mouse buttons for Yes and No, respectively. Compared with controls, meditators endorsed significantly more pleasant and fewer unpleasant words as self-referential. These two studies confirm that experienced meditators show less self-related effect (Symons and Johnson, 1997). Moreover, MRI studies (Lumma et al., 2017; Singleton et al., 2021) also indicate that meditation induces neuroplastic changes in self-referential and attentional networks from the earliest stages of the practice.

## Table S6: Meditation Related Findings Associated with Qin (2020) Model

| **Location(s)** | | **Study** | **Meditation**  **type(s)** | **Experience level (MHM)** | | | | | | | | | | |
| --- | --- | --- | --- | --- | --- | --- | --- | --- | --- | --- | --- | --- | --- | --- |
|  |  |  |  | **>10K** | **5-10K** | | | **1-5K** | | | **<1K** | | | **<30** |
| **↓ MENTAL SELF (PCC/vPcu)**  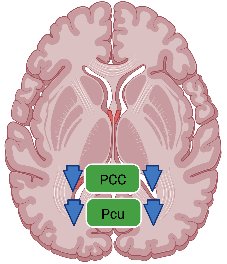 | PCC/  vPcu | Bærentsen et al., 2010 | FAM & MRM | N – 31  (MYM: 11) | | | | | | | | | | |
|  |  | Berkovich-Ohana et al., 2015 | MRM | N – 23 | | | | | | | | | | |
|  |  | Brefczynski-Lewis et al., 2007 | FAM | N – 14 | | | | | | N – 16 | | | | |
|  |  | Brewer et al., 2011 | LKM/OMM/FAM | N – 12 | | | | | | N – 12 | | | | |
|  |  | Dickenson et al., 2012 | FAM | N – 31 | | | | | | | | | | |
|  |  | Farb et al., 2007 | MBSR (FAM/OMM/LKM) | N – 20  (Training Group) | | | | | | N – 36  (No Training) | | | | |
|  |  | Garrison et al., 2013 (a) | FAM | N – 10 | | | | | | | | | | |
|  |  | Garrison et al., 2013 (b) | FAM | N – 10 | | | N – 22  N – 9 | | | | | N – 22  N – 11 | | |
|  |  | Garrison et al., 2014 | LKM | N – 20 | | | | | | N - 26 | | | | |
|  |  | Garrison et al., 2015 | OMM & FAM | N – 20 | | | | | | N – 26 | | | | |
|  |  | Hasenkamp et al., 2012 (a) | FAM | N – 9 | | | | | | N – 5 | | | | |
|  |  | Ives-Deliperi et al., 2011 | OMM | N – 10  (MYM: 4) | | | | | | | | | | |
|  |  | Lutz et al., 2016 | FAM/OMM | N – 22 | | | | | | N – 22 | | | | |
|  |  | Manna et al., 2010 | FAM & OMM | N – 8 | | | | | | N – 8 | | | | |
|  |  | Scheibner et al., 2017 | FAM | N – 20 | | | | | | | | | | |
|  |  | Simon et al., 2017 | MRM | N – 8 | | | | | | | | | | |
|  |  | Tang et al., 2013 | IBMT (FAM/OMM) | N – 33  (Meditation Training) | | | | | N – 27  (Relaxation Training) | | | | | |
|  |  | van Lutterveld et al., 2017 (a) | OMM | N – 16 | | | | | N – 16 | | | | | |
|  |  | Yang et al., 2016 | FAM/OMM | N – 13 | | | | | | | | | | |
|  |  | Yang et al., 2019 | FAM/OMM | N – 14 | | | | | | | | | | |
| **↑ EXTEROCEPTIVE SELF**  **(dlPFC, TPJ)**  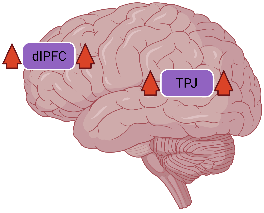 | dlPFC | Baron Short et al., 2010 | FAM | N – 8 | | | | | | N – 5 | | | | |
|  |  | Brefczynski-Lewis et al., 2007 | FAM | N – 14 | | | | | | N – 16 | | | | |
|  |  | Dickenson et al., 2012 | FAM | N – 31 | | | | | | | | | | |
|  |  | Farb et al.,2007 | FAM/OMM/LKM | N – 20  MBSR Training | | | | | | N – 36  No Training | | | | |
|  |  | Hasenkamp et al., 2012 (a) | FAM | N – 9 | | | | | | N – 5 | | | | |
|  |  | Kwak et al., 2020 | FAM/OMM | N – 23  (Meditation Training) | | | | | | N – 14  (Relaxation Training) | | | | |
|  |  | Mahone et al., 2018 | MRM | N – 16 | | | | | | | | | | |
|  |  | Manna et al., 2010 | FAM & OMM | N – 8 | | | | | | N – 8 | | | | |
|  |  | Tomasino et al., 2016 | FAM/OMM | N – 13 | | | | | | | | | | |
|  | TPJ | Bauer et al., 2019 | OMM | N – X  X = 17-Y | | N – Y  Y=17-X | | | | | | | N – 17 | |
|  |  | Dickenson et al., 2012 | FAM | N – 31 | | | | | | | | | | |
| **↑ INTEROCEPTIVE SELF**  **(INS, dACC,**  **THA)**  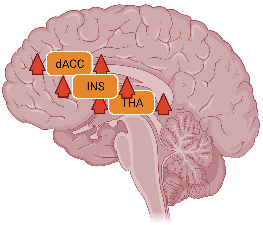 | INS | Brefczynski-Lewis et al., 2007 | FAM | N – 14 | | | | | | N – 16 | | | | |
|  |  | Engström & Söderfeldt, 2010 | MRM | N – 1  (MYM > 6.5) | | | | | | | | | | |
|  |  | Farb et al., 2007 | MBSR (FAM/OMM/LKM) | N – 20  (Training Group) | | | | | | N – 36  (No Training) | | | | |
|  |  | Farb et al., 2012 | MBSR (FAM/OMM/LKM) | N – 20  (Meditation Training) | | | | | | N – 16  (No Training) | | | | |
|  |  | Hasenkamp et al., 2012 (a) | FAM | N – 9 | | | | | | N – 5 | | | | |
|  |  | Hernández et al., 2015 | FAM/OMM | N – 23  MYM > 14.1 | | | | | | N – 23 | | | | |
|  |  | Manna et al., 2010 | FAM & OMM | N – 8 | | | | | | N – 8 | | | | |
|  |  | Tang et al., 2013 | IBMT (FAM/OMM) | N – 33  (Meditation Training) | | | | | | N – 27  (Relaxation Training) | | | | |
|  |  | Tang et al., 2015 | FAM/OMM | N – 40 | | | | | | | | | | |
|  |  | Tomasino et al., 2016 | FAM/OMM | N – 13 | | | | | | | | | | |
|  |  | Xiao et al., 2019 | MBSR (FAM/OMM/LKM) | N – 16  (Meditation Training) | | | | | | N – 16  (No Training) | | | | |
|  |  | Zeidan et al, 2013 | FAM | N – 15 | | | | | | | | | | |
|  | dACC | Hasenkamp et al., 2012 (a) | FAM | N – 9 | | | | | | N – 5 | | | | |
|  |  | Manna et al., 2010 | FAM & OMM | N – 8 | | | | | | N – 8 | | | | |
|  | THA | Brefczynski-Lewis et al., 2007 | FAM | N – 14 | | | | | | N – 16 | | | | |
|  |  | Newberg et al., 2001 | FAM | N – 8  (MYM > 15) | | | | | | | | | | |
|  |  | Newberg et al., 2010 | FAM | N – 12  (MYM > 15) | | | | | | N – 14 | | | | |

*Supporting Information: Mean Hours of Meditation (MHM). Text within sections allocated to MHM is to specify either the differentiating factor between groups of similar experience level (ex. Meditation training versus relaxation training) or the level of meditation when mean hours of experience were not provided within the published paper.*
